# Applying thermal demagnetization to archaeological materials: A tool for detecting burnt clay and estimating its firing temperature

**DOI:** 10.1371/journal.pone.0289424

**Published:** 2023-10-09

**Authors:** Yoav Vaknin, Ron Shaar, Oded Lipschits, Adi Eliyahu Behar, Aren M. Maeir, Erez Ben-Yosef

**Affiliations:** 1 Institute of Archaeology, Tel Aviv University, Tel Aviv, Israel; 2 Institute of Earth Sciences, The Hebrew University of Jerusalem, Jerusalem, Israel; 3 The Department of Land of Israel Studies and Archaeology and the Department of Chemical Sciences, Ariel University, Ariel, Israel; 4 The Institute of Archaeology, The Martin (Szusz) Department of Land of Israel Studies and Archaeology, Bar-Ilan University, Ramat Gan, Israel; New York University, UNITED STATES

## Abstract

Burnt materials are very common in the archaeological record. Their identification and the reconstruction of their firing history are crucial for reliable archaeological interpretations. Commonly used methods are limited in their ability to identify and estimate heating temperatures below ~500⁰C and cannot reconstruct the orientation in which these materials were burnt. Stepwise thermal demagnetization is widely used in archaeomagnetism, but its use for identifying burnt materials and reconstructing paleotemperatures requires further experimental verification. Here we present an experimental test that has indicated that this method is useful for identifying the firing of mud bricks to 190⁰C or higher. Application of the method to oriented samples also enables reconstruction of the position in which they cooled down. Our algorithm for interpreting thermal demagnetization results was tested on 49 miniature sun-dried “mud bricks”, 46 of which were heated to a range of temperatures between 100⁰C to 700⁰C under a controlled magnetic field and three “bricks” which were not heated and used as a control group. The results enabled distinguishing between unheated material and material heated to at least 190⁰C and accurately recovering the minimum heating temperature of the latter. Fourier-Transform Infrared Spectroscopy (FTIR) on the same materials demonstrated how the two methods complement each other. We implemented the thermal demagnetization method on burnt materials from an Iron Age structure at Tell es-Safi/Gath (central Israel), which led to a revision of the previously published understanding of this archaeological context. We demonstrated that the conflagration occurred within the structure, and not only in its vicinity as previously suggested. We also showed that a previously published hypothesis that bricks were fired in a kiln prior to construction is very unlikely. Finally, we conclude that the destruction of the structure occurred in a single event and not in stages over several decades.

## 1. Introduction

Burnt archaeological materials are common in archaeological excavations. Their identification, reconstruction of their firing history and estimation of the firing temperatures are keys to reliable interpretation of their archaeological contexts. For this, several laboratory methods have been developed. These include mineralogical studies, such as petrography and powder X-ray diffraction measurements [1], which are useful for identification of heating temperatures that exceed 600⁰C. The firing temperatures of ceramics are often determined using thermogravimetric analysis [2, 3]. Change in color is sometimes used in order to identify burnt materials and to reconstruct firing history [4–7]. Fourier-Transform Infrared Spectroscopy (FTIR) is a frequently used method that identifies the alteration of clay minerals above ~500⁰C [6–12]. Regarding lower heating temperatures, it has been shown that if ceramics are exposed to temperatures above 100–200⁰C, lipids absorbed in the ceramics do not survive, and their absence may be used for identifying ceramics that were exposed to fire [9]. However, lipids may not be identified in ancient ceramics due to poor preservation, so their absence alone cannot be used as an indication of heating. In any case, this method, which is applicable only to ceramic vessels, cannot be used for a precise estimation of the firing temperature. Another method which has been applied for the estimation of heating temperature is thermoluminescence [13–16]. Rock magnetism can also be applied for estimating firing temperature by measuring susceptibility, hysteresis parameters or isothermal and anhysteretic remanences [1, 5, 15, 17–22]. Such magnetic methods are limited in identifying materials heated to 300–400⁰C or lower and in accurately estimating these low heating temperatures [23].

The above-mentioned methods do not provide any information regarding the orientation in which the clay-based materials were heated and cooled. This can be crucial for the archaeological interpretation of the findings. For example, burnt bricks within a wall could have been fired in a kiln prior to construction [9, 24], during the destruction of the site by fire, or fired twice, during construction and destruction. To date, the only method which can identify the cooling orientation is demagnetization of oriented samples. This method has been used to reconstruct site formation processes of structures destroyed by conflagration [25–27]. Thermal demagnetization has also been used in order to examine the stability of the natural remanent magnetization (NRM) [18, 21, 22]. The use of thermal demagnetization for the reconstruction of firing temperatures was suggested by Goulpeau [28], who used it to distinguish between magnetic signals recorded when building materials were pre-fired and signals recorded when they were reheated to lower temperatures during the use of a Roman hypocaust. Similarly, Francés-Negro et al. [29] used this method for estimating firing temperatures in order to distinguish between storage vessels and cooking pots. In this paper we further develop and test this archaeomagnetic technique, suggest an interpretation method and demonstrate how it can serve as a useful complementary technique. We begin by describing the method and the results of an experiment designed to test it on laboratory-made miniature sun-dried “mud bricks” which were heated under controlled conditions. We then demonstrate the application of the method to a burnt mud brick wall from Tell es-Safi/Gath as a case study.

## 2. The suggested method for identifying burnt materials and determining heating temperature

### 2.1 Underlying principles of the method

Clays are rich in detrital ferrimagnetic minerals. The mineralogical assemblages in the clays depend on the local geology. Yet, it is common to all iron-bearing clay minerals that when they are heated to temperatures starting from ~150⁰C and up to ~700⁰C, they are transformed into stable ferrimagnetic minerals such as magnetite (Fe_3_O_4_), maghemite (γ-Fe_2_O_3_) and hematite (α-Fe_2_O_3_)—in a stochiometric form or with substitution of Ti, Mn, or Al [30–32]. The effect of the heating is twofold. First, transformation and stabilization of the magnetic mineralogy and second, acquisition of thermoremanent magnetization (TRM), proportional and parallel to the ambient magnetic field. The basic concepts of TRM relevant to the suggested method are associated with the Curie temperature (T_c_), blocking temperature (T_B_) and unblocking temperature (T_UB_). The Curie temperature is determined by the mineralogical composition and is the temperature above which the mineral loses its ferrimagnetic properties. The blocking temperature (T_B_≤T_c_) is the temperature under which TRM is acquired upon cooling from a high temperature in the presence of an ambient field. The unblocking temperature (T_B_≤T_UB_≤T_c_) is the temperature above which the TRM acquired at T_B_ is completely erased upon heating in “zero-field” conditions (in a negligible ambient field created in the paleomagnetic ovens).

The underlying assumption of the method, verified in our experiments described below, is that sun-dried mud bricks or any other archaeological dehydrated clay materials comprise weak magnetization that is significantly weaker than TRM. Thus, if a mud brick is heated to a certain temperature the previous natural magnetization is lost and the material acquires TRM. In the lab we gradually demagnetize the TRM by heating the material under zero-field conditions in a series of heating-cooling steps at progressively elevated temperatures and measure the magnetization after each step. A complete loss of the TRM is achieved at the maximum T_UB_, which can provide a close approximation of the ancient heating temperature, provided that the maximum T_B_ of the material, which depends on the magnetic mineralogy and the grain size distribution, is higher than the heating temperature. We note that in other cases, when the ancient heating temperature is higher than the maximum T_B_, we can determine only a lower boundary for the ancient heating temperature.

**Fig 1:**
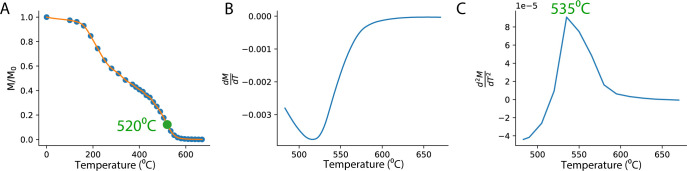
A representative example of the thermal demagnetization technique for estimating the ancient heating temperature (specimen SF12E33a- see details below). **(A)** The remaining magnetization, normalized to the initial NRM after every thermal demagnetization step (blue dots) and a cubic spline interpolation (orange line). The temperature to which the specimen was initially heated in the TRM recording stage of the experiment (520⁰C in this example) is marked in green. **(B-C)** The first (B) and second (C) derivatives of the curve presented in (A) starting after 75% of the NRM was demagnetized. The maximum of the second derivative (535⁰C) is the approximation of the ancient heating temperature.

In order to estimate the “ancient” heating temperature, we examined the remaining TRM (normalized to the initial TRM) after each demagnetization step (Fig 1A). With the rising temperatures at each of the demagnetization steps, the magnetization gradually decreases while approaching the maximum T_UB_. We estimate the maximum T_B_ by extrapolating the demagnetization data by cubic-spline and calculating the first (Fig 1B) and second (Fig 1C) derivatives of the curve. We take the maximum of the second derivative as the best estimation for the maximum T_B_. Our experience shows that in some cases the material contains two or more groups of minerals with distinctly different T_C_ and T_B_ (as in Fig 1). In these cases, we get more than one maximum in the second derivative. To generalize the method to these cases, the user defines a threshold value for the normalized magnetization, which we set by default to 0.25 and which can be changed according to the mineralogy of the studied material. The maximum of the second derivative is calculated only at the temperature interval after the normalized magnetization drops below this threshold value. A python program implementing this algorithm based on the community- standard MagIC format [33] is provided in the supplementary material (see: Extended methods in S1 Text).

The above technique does not require samples oriented in the field. However, applying this technique to oriented samples that were sampled in the orientation in which they had cooled also provides the direction of the ancient TRM. Comparison of the archaeomagnetic direction with the reference direction of the geomagnetic field provides useful information associated with movement of the material after acquiring TRM. This concept is illustrated in Section 3 below.

Finally, we suggest carrying out a set of rock-magnetic experiments in order to shed light on the magnetic mineralogy of the studied materials and provide another independent approximation of the ancient heating temperature. These include measuring saturation isothermal remanent magnetization (SIRM). Creating repeated thermomagnetic curves of magnetic susceptibility at progressively elevated peak temperatures between 100–700⁰C is useful as a means of detecting the temperature below which the material is magnetically stable. Measuring hysteresis loops and back-field isothermal remanent magnetization (IRM) and creating first order reversal curve (FORC) diagrams are useful in order to further characterize the magnetic minerals, including the domain state, which may change after heating.

### 2.2 Paleomagnetic procedures

We used a 2G Enterprises RAPID superconducting rock magnetometer (SRM) system with an in-line 2-axis AF demagnetizer in order to measure magnetic moments and carry out Alternating Field (AF) demagnetization. Thermal magnetization and demagnetization were carried out using laboratory-modified computer-controlled ASC-TD48 paleomagnetic ovens. Bulk susceptibility and thermomagnetic curves of magnetic susceptibility were measured using an AGICO MFK-1 Kappabridge with a CS4 furnace. For the thermomagnetic curves we used a heating rate of ca. 14⁰C/min. All the magnetic experiments mentioned above were carried out at the magnetically shielded paleomagnetic laboratory at the Institute of Earth Sciences, The Hebrew University of Jerusalem. Magnetic hysteresis and first order reversal curves (FORC) were measured using a Lakeshore 8604 vibrating sample magnetometer (VSM). FORC data were analyzed using FORCinel [34] and FORCtool [35] programs following the methods described in Heslop et al. [36]. Saturation isothermal remanent magnetization (SIRM) was imparted using an ASC IM-10-30 impulse magnetizer.

Demagnetization experiments were analyzed using the PmagPy Demag-GUI program [33] and displayed on Zijderveld [37] orthogonal end-point diagrams. Paleomagnetic directions were calculated using the Principle Component Analysis (PCA) approach of Kirschvink [38]. We used the Maximum Angular Deviation [MAD, see: 38] parameter to quantify the scatter of the points on the Zijderveld plots and the Deviation Angle [DANG, see: 39] to quantify convergence toward the origin. We considered demagnetization experiments with MAD>5 or DANG>5 as unreliable and in all of our analyses we rejected specimens based on these acceptance criteria. We avoided using two-component vectors for temperature estimations even if one of the components met the MAD and DANG criteria. Sample and site means with their α_95_ angle (95% confidence cone) and k (Fisher precision parameter) were calculated using Fisher statistics [40].

### 2.3 Testing the method on laboratory heated mud bricks

#### 2.3.1 Sample preparation and measurement procedures

In order to test the method, we created miniature “mud bricks” from recycled ancient mud bricks that we sampled from Tell es-Safi/Gath (Wall 19D92B06, Area D2, S1 Fig) [41]. No signs of fire were observed in the vicinity of these bricks and experiments showed that the mud bricks had indeed not been burnt in-situ and probably had not been burnt at all, at least not to temperatures higher than 190⁰C (see: S2 Fig and Extended methods in S1 Text for details). From this unburnt mud brick material we prepared 149 specimens for various experiments which are summarized in Table 1 and S1 Table.

**Table 1:** The main experiments carried out on the unheated clay.

Specimen names	No.	Crushed?	Container	Step I	Step II	Step III
SF12E01-46	46	yes	Crucibles	DhRM	In-field heating	Thermal demag.
SF12E47-49	3	yes	Crucibles	DhRM	Thermal demag.	-
SF12E50-51	2	yes	Plastic boxes	DhRM	AF demag.	SIRM
SF12E61-82	22	yes	Crucibles	In-field heating	SIRM	-
SF12E100, 130, …670, 700	21	No	Crucibles	In-field heating	AF demag.	-
SF12E000, 100, 130,…, 700	22	Yes	Capsules	Hysteresis	Back-field	FORC
SF12E000,100,310,700	4	Yes	-	In-field heating	Thermomagnetic curves	
SF12E000C, 100C, 190C, 280C, 370C, 460C, 490C, 520C, 550C, 640C, 700⁰C	11	Yes	Pellets	FTIR		

The various experiments which are referred to in the table are described in detail in the text below. The experiments carried out on this material in order to study the viscous remanent magnetization acquisition and destruction curves are described in S1 Table and in the “Extended methods” section in S1 Text.

**Fig 2:**
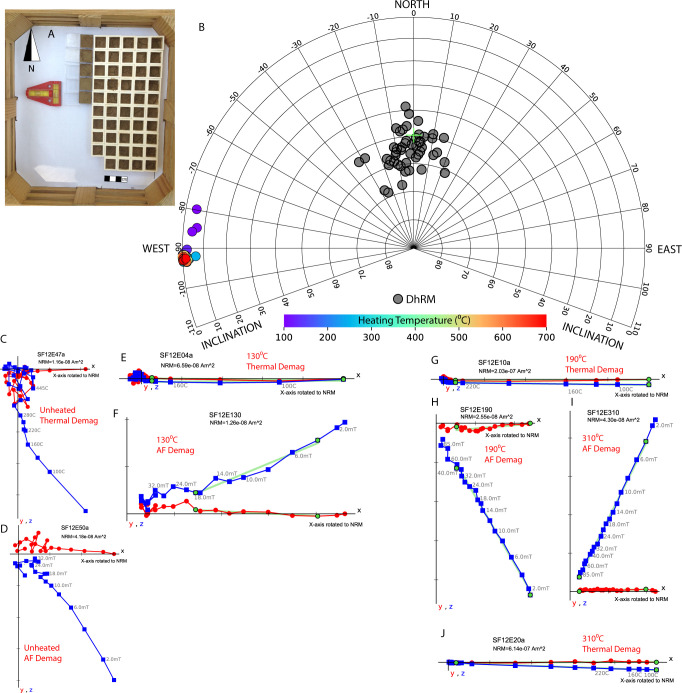
NRMs and demagnetization data of sun-dried and laboratory-heated “mud bricks”. **(A)** A picture of the experimental design showing 54 miniature “mud bricks” on a leveled tray facing north. **(B)** Equal area projection showing in gray the NRM (“DhRM”) directions of 46 sun-dried “bricks, with the direction of the ambient field marked by a green cross. The colored symbols show the NRM directions of the same 46 “bricks” after their being heated in the lab to different temperatures (see color code) in a paleomagnetic oven with a 60 μT ambient field. **(C)-(J)** Zijderveld end-point orthogonal diagrams displaying representative results of thermal and AF demagnetization experiments. Since the presented AF experiments were carried out on unoriented specimens, the x-axis is rotated to the direction of the NRM (for AF and thermal experiments). Blue and red symbols represent x–y and x–z projections of the NRMs in the specimen coordinate system, respectively. The best-fit line for specimens that met criteria is marked in green.

*DhRM*. The mud brick material was crushed using a mortar and pestle and mixed with water until obtaining a homogeneous composition which was dry enough so that a wet brick could maintain its shape without a cast (S3B Fig). Forty-nine alumina crucibles (2x2.2x2.2 cm in size), which had first been thermally demagnetized at 600⁰C (SF12E01-49), and two non-magnetic paleomagnetic plastic sampling boxes (SF12E50-51), 1.5x2x2 cm in size, were partially filled with the mud (Fig 2A). These miniature “mud bricks” were put on a horizontal wooden tray oriented facing north (S3A Fig) and left to dry for three days in a partially shaded area away from modern magnetic disturbances. After three days, we gradually dripped non-magnetic potassium silicate glue (Kasil) onto the “bricks”, a drop every several hours, without moving them. This was done in order to fill cracks in the drying mud and prevent the “bricks” from crumbling during the experiments without dissolving them in a large amount of wet glue. When the glued “bricks” had dried, we brought them into the magnetically shielded laboratory and within two hours we measured their NRM, their bulk susceptibility and their net mass (after subtracting the mass of the crucibles/ plastic boxes and the glue).

*In-field heating*. Among the 46 specimens (SF12E01-46) we heated three “bricks” at each of the low temperatures (100⁰C, 130⁰C, 190⁰C) and two “bricks” at each of the higher temperatures (220–700⁰C in increments of 30⁰C) under a controlled magnetic field. Heating the bricks was carried out in an oven field of 60μT in a direction perpendicular to the ambient field in which the bricks had been dried (“west”). Immediately after heating each group of bricks, we measured their NRMs and their bulk susceptibilities again. Three “bricks” (SF1247-49) were not magnetized in the oven at all and served as a control group. Altogether, the test included 21 groups of samples heated to different temperatures and one control group of unheated samples.

*Thermal demagnetization*. We thermally demagnetized all 49 crucible “bricks” at progressively elevated temperatures, in increments of 30⁰C from 100⁰C up to 370⁰C and in increments of 15⁰C from 385⁰C to 700°C or until the NRM was fully demagnetized. The heating times were 40 min. in the 100–160⁰C steps, 50 min. in the 190–310⁰C steps, 60 min. in the 340–370⁰C steps and 65 min. in the 400–700⁰C steps.

*AF demagnetization*. AF demagnetization experiments were carried out on two unheated miniature “bricks” prepared in plastic boxes (SF12E50-51). In addition, we prepared 21 unoriented specimens (SF12E100, 130, …670, 700) by heating 21 pieces from the uncrushed mud brick material, one to each of the 21 temperatures used in the experimental test (100–700⁰C in increments of 30⁰C) in an oven field of 60μT. All AF experiments were carried out at progressively elevated peak fields in 2mT steps up to 20mT, 4mT steps up to 40mT, 10mT steps up to 70mT and 15mT steps up to 100mT.

*SIRM*. An additional set of 22 specimens (SF12E61-82) was prepared in crucibles from the same crushed material and Kasil glue (in the shielded room and with no water). Every one of these specimens was given a TRM as mentioned above. After measuring its TRM we magnetized every specimen to saturation isothermal remanent magnetization (SIRM) in a 1.4T field and measured this magnetization within 2 minutes.

*Hysteresis*, *back-field and FORC*. We prepared 22 specimens for magnetic hysteresis, back-field curves and first order reversal curves (FORC). One of these specimens was prepared from the unburnt mudbrick material (SF12E000) and the remaining 21 were prepared from mud brick material which had been heated in the lab to 100–700⁰C in increments of 30⁰C. The FORC curves were measured using regularly-spaced 450 loops with -50mT<Bu<0.5mT and Bc <100 mT.

*FTIR*. FTIR spectrometry was measured on eleven samples: one sample was prepared from the clayish material used to prepare the experimental miniature “bricks” and ten samples were prepared from the same material after heating, each to one of the following temperatures: 100⁰C, 190⁰C, 280⁰C, 370⁰C, 460⁰C, 490⁰C, 520⁰C, 550⁰C, 640⁰C or 700⁰C. Using a press, we then obtained 5 mm-diameter pellets, each containing ~0.1mg of material from one of the samples mixed for dilution with KBr (IR Grade). FTIR was measured in the Department of Chemical Sciences at Ariel University using a Thermo iS5 spectrometer. Spectra were measured from 4000cm^-1^ to 400cm^-1^ at a resolution of 4cm^-1^.

#### 2.3.2 Results

**Fig 3:**
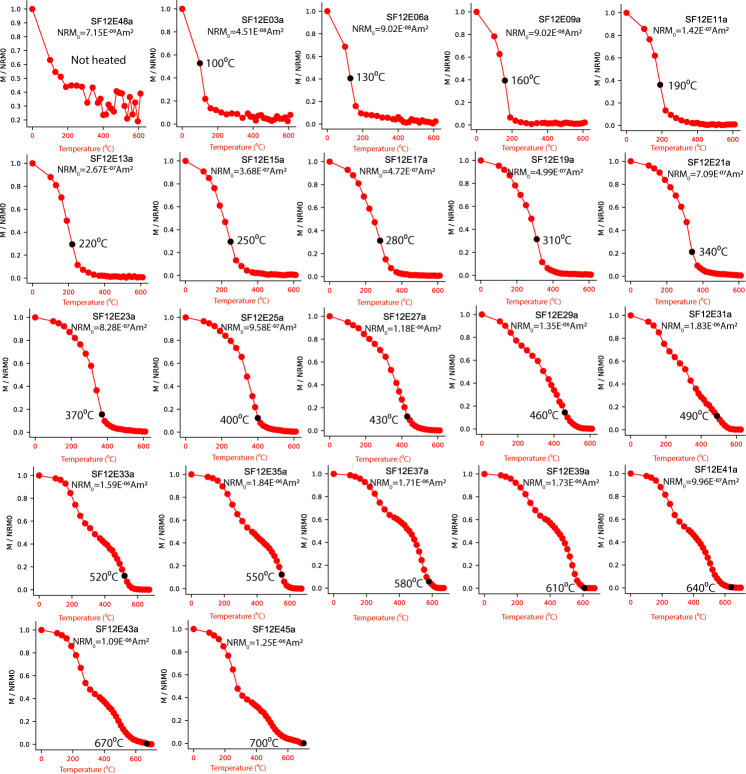
Representative results of thermal demagnetization experiments. Each sub- figure displays remaining magnetization after every demagnetization step, normalized to the initial magnetization (NRM) versus the temperature of the demagnetization step. The NRM of the unheated specimens (top- left sub- figure) is the DhRM recorded outdoors. The NRM of the other specimens is the magnetization recorded in the paleomagnetic oven (marked by the black circle).

While drying in the sun, all 51 specimens (SF12E01-51) recorded NRM in a direction roughly parallel to the direction of the ambient geomagnetic field. In Fig 2B we show for comparison the results of 46 specimens after drying in the sun (grey circles) and after being heated in an oven with a lab magnetic field (color-coded). The Fisher mean of the 46 NRMs points to the direction of the ambient geomagnetic field: declination = 357, inclination = 55, α95 = 2.3, circular standard deviation (CSD) = 8.9, *k* = 81. The nature of this recorded magnetization is partly viscous but also consists of a significant component associated with dehydration (see below). We therefore tentatively refer to it hereafter as “dehydrational remanent magnetization” (DhRM). Representative demagnetization data of the DhRM are displayed in Fig 2C, 2D and in Fig 3, showing relatively straight-line Zijderveld diagrams. Yet, these lines do not converge well to the origin and out of five non-heated samples, only one passed the Maximum Angular Deviation (MAD) and Deviation Angle (DANG) criteria (Section 2.2), indicating poor stability of the “DhRM”. The results of the demagnetization measurements of SF12E are available in S1 File.

The directions of the TRMs of the 46 specimens are tightly clustered around the reference laboratory direction (declination = 270, inclination = 0), with the slight exception of the three specimens which were magnetized at 100⁰C (Fig 2B). The Fisher mean of the archaeomagnetic directions calculated by PCA, after excluding five specimens that failed the MAD and/or DANG criteria is: declination = 268, inclination = 1, α_95_ = 0.7, CSD = 2.9, *k* = 785. The small α_95_ and high *k* indicate high precision recording of the TRM. We note that the five specimens failing criteria were heated to temperatures below 190⁰C. Fig 2E–2J shows examples of demagnetization data for specimens heated to low and moderate temperatures (130⁰C, 190⁰C, 310⁰C), demonstrating that the magnetic signal becomes more unified as the heating temperature increases.

**Fig 4:**
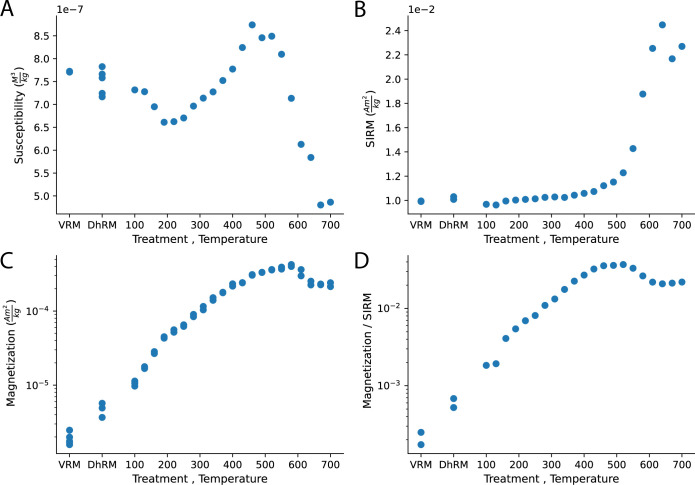
Bulk magnetic properties versus treatment (VRM or DhRM) or heating temperature. **(A)** Mass normalized susceptibility. **(B)** Mass normalized Saturation Isothermal Remanent Magnetization (SIRM). **(C)** Mass normalized magnetization. **(D)** Magnetic moment normalized to SIRM.

The gradual change in the magnetic properties of the heated mud is displayed in Fig 3, which shows normalized magnetization during thermal demagnetization, Fig 4, which shows bulk susceptibility, magnetization and SIRM, S4 Fig, which shows the hysteresis parameters as well as several representative FORC diagrams and S5 Fig that shows thermomagnetic curves. It is evident from Fig 3 that increasing the heating temperature expands the T_UB_ spectrum and that the maximum T_UB_ is typically higher than the heating temperature. In addition, the magnetization (whether it is partial TRM, TRM, or thermochemical remanent magnetization in origin), the SIRM and the magnetization normalized to the SIRM increase up to ~550⁰C (Fig 4B–4D). The susceptibility change (Fig 4A) is more complicated but shows a steady increase between 190⁰C– 460⁰C. Altogether, this indicates that heating the mud to temperatures up to 520⁰C enhances the capacity of the samples to acquire magnetization due to formation of new ferromagnetic minerals. At higher temperatures the picture is more complex as the susceptibility shows a fast drop above ~520⁰C, the SIRM slightly decreases above 650⁰C and the magnetization decreases above 610⁰C. The thermomagnetic curves (S5 Fig) explain this behavior by a gradual formation of magnetite and/or maghemite up to 550⁰C and formation of weaker hematite at higher temperatures [42, 43], although the contribution of the hematite to the magnetization (Fig 3) is suppressed by the magnetite. The hysteresis (S4A and S4B Fig) and the FORCs show that the main change in the coercivity spectra and Mr/Ms ratio occurs above 520⁰C, with a change from samples dominated by pseudo-single domain and superparamagnetic particles (S4C–S4F Fig) to non-interacting single domain (S4H Fig).

Figs 3 and 4 show that the source of the magnetization of the sun-dried samples is not entirely viscous and that there is a significant component associated with dehydration. The fact that there is no significant difference in susceptibility and SIRM between the VRM and the DhRM samples (Fig 4A and 4B) indicates similar magnetic mineralogy. However, the magnetization of the DhRM samples is ca. 2–3 times stronger than that of the VRM samples (Fig 4C and 4D), implying a mechanism associated with the physical stabilization of minerals within the bricks while they were being sun-dried rather than solely VRM. To further understand the role of VRM in our experiments, we show in S6 Fig the processes of acquisition and destruction of VRM.

**Fig 5:**
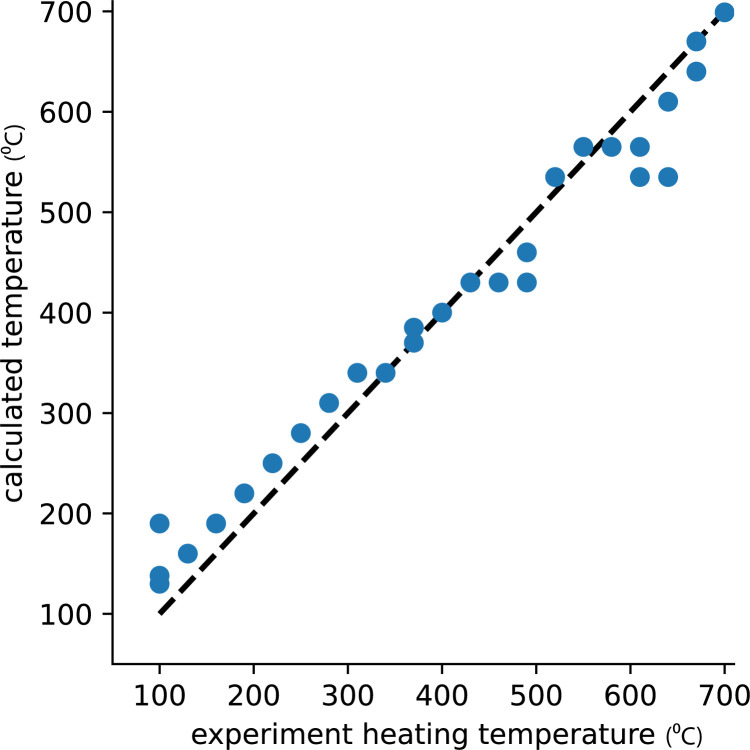
Calculated heating temperature versus true heating temperature. For every specimen we display the actual heating temperature which was carried out in the lab (x axis) and the estimated heating temperature (y axis) according to the suggested method (as in Fig 1). For each temperature the results of all 2–3 specimens are displayed and, in most temperature steps, they overlap completely. The black dashed line is the 1:1 ratio between the calculated and the true heating temperatures.

Based on the magnetic characterization data described above, we argue that this set of samples is suitable for testing the method for reconstructing ancient heating temperature. Using the algorithm described in section 2.1 to recover the heating temperature resulted in very good agreement between the actual heating temperatures and the estimated “ancient” heating temperatures for all 46 specimens (Fig 5). This demonstrates the success of the method for the clay material used in our experiments. We note that the method slightly overestimates the heating temperature below 300⁰C and slightly underestimates it above 450⁰C. The average error for heating temperatures lower than 610⁰C was up to 45⁰C and for heating temperatures of 610–700⁰C it was up to 70⁰C.

**Fig 6:**
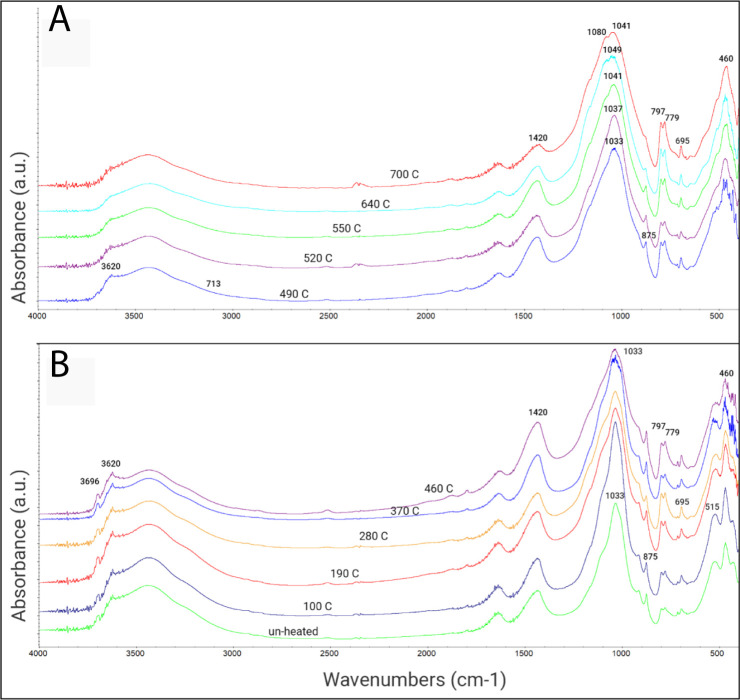
FTIR results of experimentally heated mud brick material. **(A)** FTIR spectra of mud brick material after every specimen was heated in the lab to a temperature in the range of 490–700⁰C. **(B)** FTIR spectra of unheated mud brick material and the same material after it was heated to temperatures in the range of 100–460⁰C, showing no significant changes.

The ability of the magnetic algorithm to accurately recover the heating temperature is now compared to the results of the FTIR method. Representative FTIR spectra of the mud brick material before and after being heated in the lab to increasing temperatures are shown in Fig 6. According to the spectrum of the unheated mud brick (Fig 6B), clay (main peaks at 3625, 3427, 1033, 914, 518 and 468 cm^-1^), calcite (1420, 875 and 713cm^-1^) and quartz (1084, 797–779 doublet, 695, 515 and 460cm^-1^) are the three main mineralogical constituents. The clay fraction is dominated by montmorillonite clay type (the TOT smectite group) with minor amounts of kaolinite [8, 11]. In general, our results are in agreement with previous FTIR measurements of archaeological mud bricks and experimentally-heated sediments [e.g. 8, 11]. Up to 460⁰C, changes in the structure of most clay minerals are not expected and the corresponding spectra are indeed almost identical to the spectrum of the unheated material (Fig 6B). In the 460⁰C spectrum, the absorptions of the bounded hydroxyls are still visible, suggesting that dihydroxylation had not occurred. Thus, using only FTIR, this material would not be identified as burnt or exposed to heat. Only heating temperatures in the range of 490–700⁰C show significant structural transformations, with a weakening of the Si-O-Al absorption at ~518cm^-1^ and of the Al-O-H absorption at ~914cm^-1^ as manifested by the FTIR spectra (Fig 6A). Furthermore, the rising temperature generally resulted in an increasing shift of the main Si-O-Si vibration towards higher wavenumbers from 1033 to 1041cm^-1^. For more details, see Extended results in S1 Text.

At 400⁰C, roughly 100⁰C lower than the temperature at which a clear change was noticed in the FTIR spectra, reddening of the color of the bricks could be identified (S7 Fig) as previously published [8]. In our experiments we have also noted that in accordance with the color change, all samples of unheated brick material and those heated to 400⁰C or lower immediately disintegrated in water (S7B Fig).

## 3. Case study: A burnt mud brick wall and collapsed material in Area A in Tell es-Safi/Gath

### 3.1 Archaeological background

Tell es-Safi is one of the largest archaeological mounds in central Israel. It is located ca. 40km west-south-west of Jerusalem and identified with biblical Philistine Gath. Excavations at the site have revealed a well-defined destruction layer with extensive evidence of a site-wide destruction, with massive burning, in a well-defined chronological horizon [41]. This destruction is dated to ca. 830 BCE (based on radiocarbon and pottery seriation) and is linked to the siege and destruction of Philistine Gath by Hazael, King of Aram Damascus, as mentioned in II Kings 12:18. One specific part of this destruction layer in Area A, Wall 123007 and its vicinity (Square 89C, Stratum A3) was previously examined using macro- and micro-archaeological tools [9]. Based on the uniform heating pattern of mud bricks from Wall 123007, Namdar et al. [9] concluded that these bricks had been pre-fired prior to construction of the wall. Based mainly on the absence of alteration of clay minerals in FTIR spectra in samples from the lower surfaces, including a phosphate-rich ash layer found on the floor of the structure, and on the presence of lipids in ceramics found on the floor, they concluded that the conflagration had occurred in the vicinity of the structure and on its roof but not within it. According to their interpretation, the phosphate-rich ash layer was produced outside the structure and was redistributed by the wind into the structure. Based on the stratigraphic order of the surfaces within the destruction layer, Namdar et al. [9] reached the conclusion that the roof must have survived the fire without immediate collapse and that it collapsed only decades after the conflagration. We returned to this archaeological context (labeled hereafter SF09) to re-examine it using the tool we developed. The archaeomagnetic directions measured from 74 specimens from SF09 were mentioned briefly in Vaknin et al. [44] where they are labeled as “Gath_structure” and “Gath_collapse_Area_A”. Here we present new results measured from 182 additional specimens sampled from the same context in order to identify heating of clay, to estimate firing temperatures and to reconstruct cooling orientations.

### 3.2 Archaeomagnetic experiments

**Fig 7:**
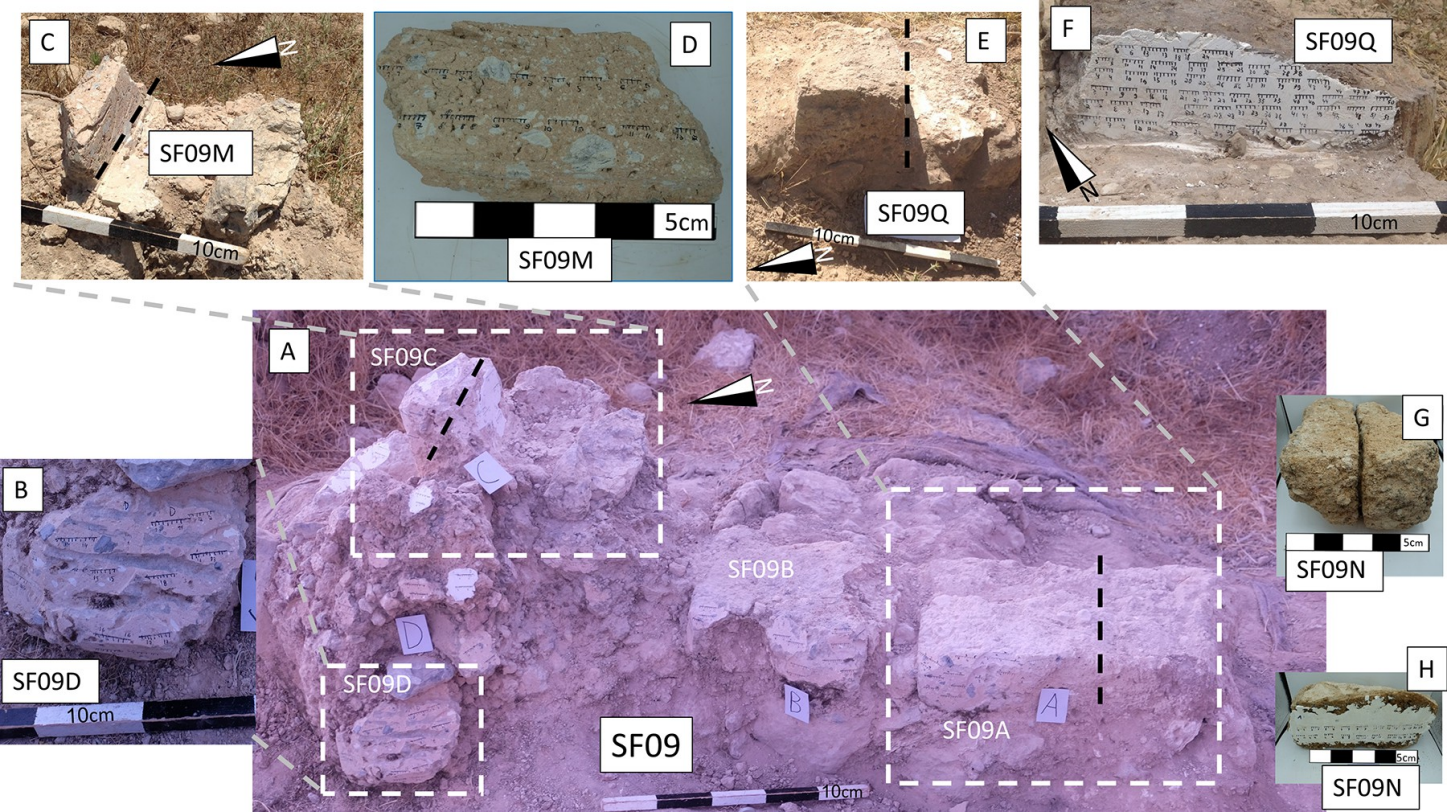
Representative samples taken from Wall SF09 in Tell el-Safi/Gath. **(A)** A general view of the wall and its vicinity. **(B)** A zoom-in view of a consolidated sample (labeled here SF09D). Thin branch impressions are visible on this sample and it most likely originated from the roof (SF09D is marked by an arrow in Fig 3d in Namdar et al. [9]). **(C-D)** A section through brick SF09C, labeled SF09M. The location of the section is marked by a black dashed line in (C) and in (A). **(E)** A section through brick SF09A, labeled SF09Q, after its southern part was removed. The location of the section is marked by a black dashed line here and in (A). **(F)** Section labeled SF09Q in brick SF09A. **(G-H)** A section in brick SF09N which was removed during the excavation. See S8 Fig for a photo of the other side of SF09 where seven additional samples can be seen.

We sampled 13 oriented “hand samples” [45] from in-situ bricks in the wall (SF09A-B in Fig 7A, S8 Fig) and from collapsed materials around the wall (SF09C-K in Fig 7A and 7B, S8 Fig) and one unoriented almost intact brick (SF09N) which was removed during the excavation (Fig 7G and 7H). We designated every brick or group of collapsed material as a separate sample. In the lab we cut a section through SF09N (Fig 7G and 7H). In the field we cut a section through the middle of one in-situ brick (SF09A) after hardening part of it with Kasil glue and labeled it SF09Q (Fig 7E and 7F). In the field we also cut a section through a roof fragment or collapsed brick (SF09C), which seemed to be consolidated by the fire, and labeled it SF09M (Fig 7C and 7D). In order to create flat surfaces needed for accurate orientation measurements, in some cases we added a thin layer of Plaster of Paris (Fig 7, S8 Fig).

After creating flat surfaces, we marked horizontal lines (Fig 7) and measured their orientation using both a magnetic Brunton compass and a sun compass, following the methods described in Vaknin et al. [44]. After removing the samples we cut them into specimens while maintaining their orientations. The specimens were glued in thermally demagnetized alumina crucibles for thermal demagnetization or in paleomagnetic plastic sampling boxes for AF demagnetization. We also measured FTIR spectrometry on 14 specimens from the inside of two bricks from this context: five from SF09M and nine from SF09Q. Since we applied Kasil glue to SF09Q for the archaeomagnetic sampling, the samples for FTIR were taken from an additional section made in this brick where glue was not applied.

All necessary permits were obtained for this study, which complied with all relevant regulations. All the archaeological samples mentioned in this paper were excavated under license number G-41/2022 by the Israel Antiquities Authority. All samples are stored in the archaeomagnetic laboratory at Tel Aviv University and are available for study. Specimens from all these samples that were measured in the different experiments are stored in the paleomagnetic laboratory at the Institute of Earth Sciences, the Hebrew University of Jerusalem and are available for study as well.

### 3.3 Case study results

**Fig 8:**
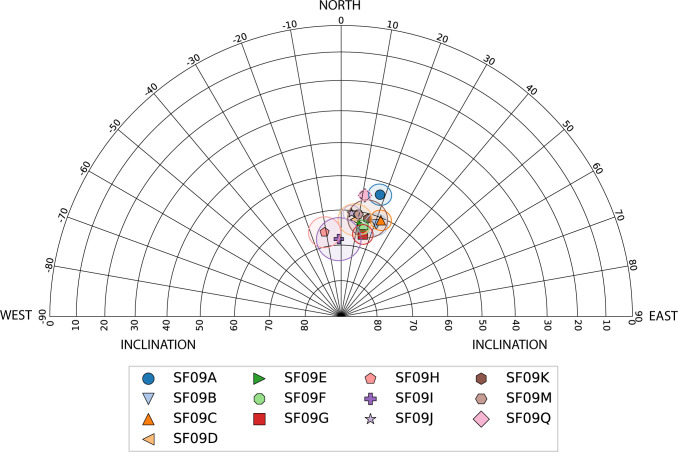
All archaeomagnetic directions of oriented samples collected from SF09 (see: Fig 7, S8 Fig). For every specimen, the calculated direction is based on the best-fit line of the AF or thermal demagnetization experiment. After rejecting specimens which failed the MAD and DANG criteria we calculated for every sample its Fisher mean (marked by a colored symbol) and its α_95_ confidence cone (marked by a circle). These results are presented also in S4 Table.

We carried out AF and thermal demagnetization (S2 and S3 Tables) on 256 specimens, 214 of which were prepared from 13 oriented samples collected from the wall and the collapsed material around it. 203 of the 214 oriented specimens met the selection criteria (MAD≤5, DANG≤5). All 11 specimens which failed criteria are from SF09Q (S9 Fig and Extended results in S1 Text). Fig 8 and S4 Table display mean directions of the 13 oriented samples with their α_95_ confidence cones. All 42 specimens from the unoriented brick (SF09N) which underwent thermal (S10A Fig, S5 Table) and AF (S10B Fig) demagnetization met criteria (MAD≤1.6 and DANG≤0.6 for all specimens) and yielded tightly clustered directions (α_95_ = 0.6. *k* = 1229). The results of the demagnetization measurements from SF09 are available in S2 File.

**Fig 9:**
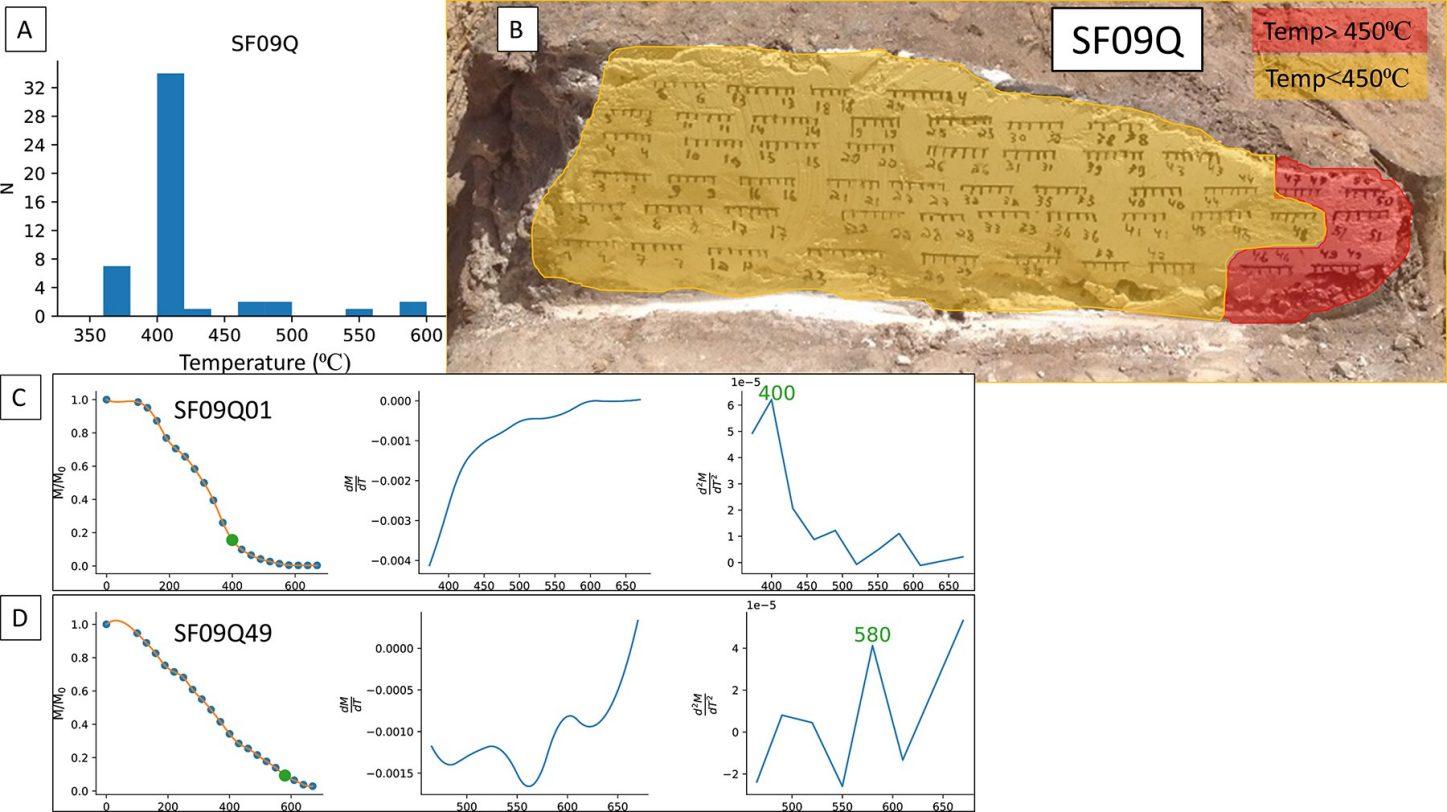
Estimated heating temperatures of SF09Q. **(A)** Histogram of calculated heating temperatures of all the specimens from the section we cut in the in situ brick (SF09Q). **(B)** Sample SF09Q showing areas with estimated minimum temperature below 450⁰C (yellow) and above 450⁰C (red). **(C)** A representative calculation of the ancient heating temperature resulting in a relatively low temperature (400⁰C). **(D)** A representative calculation of the ancient heating temperature resulting in a relatively high temperature (580⁰C).

The estimated heating temperatures of all thermally-demagnetized specimens from SF09 (Fig 9, S11 and S12 Figs) show that the temperatures within every brick were usually homogenous, with the exception of the eastern part of SF09Q (Fig 9A, 9B and 9D). Two of the sections (SF09M-N) yielded only temperatures in the range of 550–600⁰C (S11 and S12 Figs). Only section SF09Q yielded mainly lower temperatures, in the range of 350–450⁰C with the exception of 5 specimens, all in the eastern outer part of the brick, which were heated to temperatures in the range of 460–580⁰C.

The FTIR spectra of SF09M shows signs of alteration, typical of clay heated to 500–600⁰C (S13 Fig). The spectra from all specimens from SF09Q show no signs of alteration of the clay minerals (S14 Fig).

## 4. Discussion

### 4.1 Re-examining the site formation processes at Tell es-Safi/Gath, Area A

Our direction results from the outside of two in-situ bricks (SF09A-B) and from the inside of one of them (SF09Q) clearly show that after these bricks had been heated, they cooled in their original orientation within the wall, the same orientation in which they were unearthed. This heating and cooling resulted in a strong and usually unified magnetic signal, in the direction of the ancient field (Fig 8), which is similar to the average direction of the geomagnetic field in the region. Furthermore, the direction results from all the collapsed material of SF09 are also generally clustered together in the same direction (Fig 8). The slight differences between the direction results of the different samples seem to be the result of slight reorientation of these samples after they had cooled down. Such movements are expected in the case of collapsed material [27]. Indeed, the direction results of two samples of collapsed materials unearthed on the eastern side of the wall, SF09H and SF09I (S8 Fig), show lower declination than all other samples. In any case, the relatively unified directions of the magnetic signals recorded in all 13 samples, including three samples of in-situ bricks and 10 samples of collapsed material from the roof or upper courses of the wall, unearthed at different elevations and orientations, are indicative of a structure that collapsed during the fire when these materials were all still hot [27]. According to the impressions of branches left on its surface, SF09D clearly originated from the roof (Fig 7). The other nine collapsed samples originated either from the roof or from upper parts of the wall. In any case, the part of the roof supported by the wall would have clearly collapsed immediately after the collapse of these samples. Therefore, our results indicate that at least in the studied area, the structure collapsed during the conflagration, probably due to the failure of wooden beams and branches which had supported its roof.

Our results from the three sections made in bricks (SF09M, N, Q) point to a mostly homogenous temperature within every tested material, with the exception of the 50–150⁰C increase on the outer and eastern edge of SF09Q. The combination of archaeomagnetic directions and the suggested interpretation method for reconstructing firing temperatures presented here indicate that the bricks in Wall SF09 were burnt for the first time during the destruction by fire. The direction results from the wall, from the collapsed material around it and from the sections in the bricks indicate that after having been heated they all cooled down roughly in the orientation in which they were unearthed, with the possibility of only slight movement (Fig 8). Our results from the section (labeled SF09Q) through the in-situ brick (SF09A) are of special importance for this issue. Since the eastern outer part of SF09Q recorded temperatures of up to 600⁰C (Fig 9D), as did other bricks in the same wall, the magnetic minerals in these bricks can record such high temperatures. This is due to the presence of magnetite, as discussed above. Furthermore, the FTIR spectra of the inner parts of SF09Q also show that its interior was not heated to 500⁰C or more (S14 Fig). Therefore, the calculated firing temperature of the inner part of SF09Q (370–400⁰C) is not only the minimum heating temperature but also the maximum. If SF09Q had been pre-fired to ~600⁰C or more, this would be clear on the FTIR spectra. Furthermore, heating it again during the conflagration to lower temperatures (clearly in a different orientation from that in the kiln) would have resulted in two component vectors in the demagnetization experiments of many of the specimens. Eleven out of 49 specimens from SF09Q which underwent AF or thermal demagnetization failed criteria but only three resulted in clear two-component vectors (S9B–S9D Fig). The low-temperature components of these specimens are roughly in the direction of all other specimens and were clearly recorded during the conflagration. If the high-temperature components of these specimens, all from a single brick, had been in the same direction, they could have been recorded when this brick was pre-fired in a kiln. However, the high-temperature components of these specimens are in completely different directions from each other, ruling out the possibility that they were recorded by firing this brick in a kiln. These high temperature components can be explained by a small amount of burnt materials (such as small pottery fragments) or other magnetic materials which were mixed by chance into the mud composition when this brick was prepared. It is important to note that mudbrick recycling was a very common practice in the period in question, so pottery fragments could have easily been part of the mudbrick matrix. In all other 12 samples of SF09, it is clear that the magnetic moment we measured was recorded in-situ, but it is hard to rule out that the conflagration completely erased a magnetic signal which theoretically could have been recorded in a kiln. But in the case of SF09Q, it is very unlikely that it was fired in a kiln to ca. 400⁰C or less, since such low temperatures would not have consolidated the bricks and would thus have been useless. Indeed, after being heated to ca. 400⁰C by the conflagration, SF09Q tended to crumble and we had to apply glue in order to sample oriented samples for archaeomagnetism.

Although our results are in agreement with some of the previous analyses of the same structure, there is a significant discrepancy between our results and the interpretation which was suggested by Namdar et al. [9]. As shown in previous research [8] and corroborated by our results, the absence of alteration in the FTIR results is indicative not only of unheated samples, but also of *heated* samples that did not reach ~500⁰C. If the fire had taken place only on the roof of the structure and not within it [9], the bricks which were unearthed in situ, as part of an inner wall of the structure (SF09A-B), would not have been heated to 400–600⁰C since heat from fire on the roof would have been expelled to the environment and would not have heated the chamber below it to such high temperatures [7]. Therefore, the cooling of in-situ bricks within the wall after they were heated to 400–600⁰C indicates that there was a fire in the structure itself and not only in its vicinity or on the roof. Thus, it seems likely that the ash found on the floor was the result of this fire and was not redistributed into the structure by the wind, as has been suggested [9]. There was no combustion facility within the structure which could have been the source of the ash and in any case there were clear signs of destruction by fire in the entire area.

The homogeneity of the heating within bricks from this context which we found by creating sections in three bricks, was already noted in the paper by Namdar et al. [9], where it led to the conclusion that “this uniform heating pattern indicates that the bricks were pre-fired prior to being placed in a wall”. In a later study, this interpretation was considered unlikely but it could not be ruled out by the methods used [6]. The homogenous firing of mud bricks was explained there as a result of the addition of chaff to the brick composition. It was shown that in some cases, depending on the amount of chaff and the duration of the fire, the temperature in the core of a brick may even exceed that of its surroundings. In yet another study, the use of archaeomagnetic directions was suggested as the only tool which can help distinguish between pre-fired bricks and bricks fired during conflagration [26]. However, it was still hard to rule out the possibility that pre-fired bricks were heated again during the conflagration to temperatures that exceed the blocking temperature of the main magnetic minerals in the bricks. The combination of archaeomagnetic directions and estimating heating temperatures presented here, can rule this possibility out, especially when dealing with materials heated to relatively low temperatures, such as SF09Q.

The relatively low temperatures measured in SF09Q and the lack of alteration in the clays in SF09Q and in the low-elevation surfaces measured with FTIR in Namdar et al. [9] should be explained by the low elevation within the burning structure, since the floor and low courses may be heated to temperatures significantly lower than the roof and higher courses during conflagration [7]. SF09Q was several courses above the floor level (Fig 7A), where the temperature could have been even lower, perhaps even less than 200⁰C [7], which can explain the preservation of the lipids which were identified in pottery from the floor of the structure using residue analysis [9].

The direction results, combined with the other results, enable us to reconsider another aspect of this destruction: the length of time it took for the structure to collapse and for the destruction horizon to form. Namdar et al. [9] identified consolidated materials as originating from the roof of the original structure, such as SF09D mentioned above. Our results reinforce this observation. SF09C, which we identified as originating from the roof or a high course of the wall, and SF09D were consolidated by the heat. The section in SF09C (labeled SF09M) showed that it had been heated to a minimum temperature of ~550–600⁰C throughout its interior, which is in agreement with the FTIR spectra (S13 Fig). Since the recording of the magnetic signal occurs while the material is cooling down, if the roof had survived the fire without immediate collapse and collapsed over a period of decades, as has been suggested [9], we would expect random direction results from the collapsed material. Our direction results (Fig 8) strongly indicate that at least in the vicinity of the studied wall, the structure collapsed during the conflagration and not decades later.

In summary, by detecting heating of mud bricks to relatively low temperatures, lower than ~500⁰C (the detection limit of FTIR) and reconstructing paleomagnetic directions, we were able to show that: 1) The fire occurred within the studied structure (and not only in its vicinity). 2) The structure collapsed during the destruction event (and not over a period of decades). 3) The structure was built using sun-dried mud bricks (and not pre-fired bricks). This last conclusion is of outmost significance, as it relates to the broader discussion regarding mud-brick construction practices in the ancient Near East. Pre-fired bricks were surely in use in the Southern Levant during the Roman period [e.g. 46] but suggestions regarding earlier use of such bricks in this region should be reevaluated, especially if they are based mainly on FTIR [e.g. 24, 47].

### 4.2 The suggested interpretation method in a wide perspective

As demonstrated above in the case of the destruction at Tell es-Safi/Gath, treating materials burnt to temperatures lower than 500⁰C as unburnt can lead to misleading conclusions regarding the interpretation of the archaeological context. Thermal demagnetization, which is well established in archaeomagnetic research for different uses, is the basis for our proposed technique for identifying these materials and reconstructing their firing temperature. We systematically tested our technique on mud bricks, but it can be implemented on other burnt materials containing clay, such as ceramics and combustion installations (ovens, kilns, etc.).

The recording of the ambient magnetic field by unheated sun-dried bricks, as indicated by our results (Fig 2B–2D), can be useful for site formation research, although it is still challenging to distinguish between the magnetic properties of such bricks and bricks heated to temperatures lower than 190⁰C. The “dehydrational remanent magnetization” (DhRM) recorded in the dried bricks is approximately in the direction of the ambient field (Fig 2A), but the demagnetization experiments indicate low stability (Fig 2C and 2D). Even at very low heating temperatures (<190⁰C), TRM becomes dominant (Fig 2B) and at higher temperatures the TRM is orders of magnitude stronger than the DhRM (Fig 4C). Thus, we conclude that the magnetization acquired by the unheated sun-dried bricks is negligible compared to the TRM, even at low temperatures.

The basis for the method suggested here is that the temperature at which the magnetic signal is acquired (T_B_) is lower but close to the temperature at which it is erased (T_UB_) (Fig 3). It enables accurate reconstruction of the heating temperature in the range of 190–700⁰C and results indicating 100–160⁰C should be treated with caution. We would like to emphasize that the calculated heating temperature can be used only as a lower bound of the ancient heating temperature as it is possible that the maximum Curie temperature in the material is lower than the ancient heating temperature. Thermomagnetic curves are useful in order to identify such cases.

The use of thermal demagnetization for identifying burnt clay materials and accurately estimating the heating temperature is unique in its ability to deal with clay materials heated to ~190–500⁰C. In future implementations of this method we recommend measuring also the bulk susceptibility after every demagnetization step in order to combine the interpretation method suggested here with the method presented in Rasmussen et al. [1]. Other methods have different advantages. For example, immersing samples in water to test whether or not they disintegrate is a useful tool which can easily be applied in the field for initial identification of materials burnt to ~400⁰C or higher (S7 Fig), approximately 100⁰C lower than the detection limit of FTIR. Color change can also be used for preliminary identification of burnt materials but one should keep in mind that color depends on various factors, such as the initial minerology and atmosphere during firing and not only the maximum temperature [5–7]. Our method covers the lower temperature range (>190⁰C), but it will always yield minimum estimated heating temperatures of 600–700⁰C (or lower), even for materials heated to higher temperatures, due to the Curie Temperatures of the ferromagnetic minerals common in archaeological materials. The reversibility of repeated susceptibility-temperature curves at elevated temperatures can be useful in identifying materials heated to temperatures between 100⁰C to 700⁰C but the results are not always conclusive and depend on the material properties. FTIR can identify materials burnt to ~500⁰C and above but is limited in precisely estimating heating temperatures in the range of 500–700⁰C according to our results (see: Extended results in S1 Text) and previously published studies [6]. FTIR has been shown to be efficient in estimating temperatures in the range of 700–1100⁰C [8, 48] way beyond the range of archaeomagnetic thermal demagnetization.

The archaeomagnetic technique we propose, combined with the well-established method of reconstructing archaeomagnetic directions for site formation research [e.g. 27], is a powerful tool for archaeology. Combining archaeomagnetic tools with the other methods discussed throughout the text can enable new insights regarding the firing history of clay materials burnt to an unprecedented large range of temperatures. The unique advantages of our suggested interpretation method were demonstrated in the study of Area A in Tell es-Safi/Gath and can lead to reliable interpretations of clay-based burnt archaeological materials.

## 5. Conclusions

Here we show that: (1) Thermal demagnetization can identify clay materials which have been burnt to 190–700⁰C and can accurately estimate their minimum heating temperature; (2) Combined with directional analysis of oriented samples, the suggested interpretation tool is crucial for accurate reconstruction of fire-related destruction processes of mud brick structures, providing information regarding their scale and duration; (3) The studied structure was built using sun-dried mud bricks (and not pre-fired bricks), which were burnt only during the conflagration that occurred within the structure (and not only in its vicinity), resulting in the immediate collapse of the structure. (4) The common understanding that in the Southern Levant, sun-dried mud bricks and not pre-fired bricks were used for construction prior to the Roman period is most probably correct. Recent suggestions regarding earlier use of pre-fired bricks may be the result of the inability of FTIR to detect low firing temperatures.

## Supporting information

S1 Fig Location of unburnt sampled mud bricks in Tell es-Safi/Gath, Area A. **(A)** A profile view of Wall 19D92B06 (view looking south), with east side clean but west side still abutted by fill. **(B)** An aerial photo of Squares 92B, 93A and 93B in Area D2. Wall 19D92B06 is marked by a green dashed rectangle in both (A) and (B). **(C)** Oriented samples of in-situ mud bricks (SF12A01-04; close up view, looking south).
(TIF)

S2 Fig AF results of SF12A. **(A-D)** Zijderveld [37] end-point orthogonal diagrams of all 4 specimens from oriented samples from an in-situ mud brick, which is assumed to be unheated. This material was used to create the miniature mud bricks. The red circles are the projections on the north-east plane whereas the blue squares are the projections on the east-down plane. Best-fit lines and the chosen AF bounds are marked in green. The initial NRM is given below the specimen name. The MAD and DANG angles of the chosen Best-fit line are displayed as well. Since the MAD or the DANG in (A) and (D) exceed 5, these two specimens fail criteria. Demagnetization data were analyzed using the Demag GUI program, where best-fit paleomagnetic directions were calculated using principal component analysis [38]. We chose the AF bounds which represent a significant amount of the NRM and which yielded the minimum MAD and DANG. **(E)** All four directions of SF12 (including the two which failed criteria) plotted on an equal area projection.
(EPS)

S3 Fig Preceding the miniature “mud-brick” preparation. **(A)** The wooden tray placed horizontally in the field away from modern magnetic disturbances and oriented facing to the magnetic north using a Brunton compass. **(B)** An experimental mud brick made from the same mixture of crushed material and water as the miniature “bricks” in order to make sure that the brick maintains its shape without a cast.
(TIF)

S4 Fig Representative hysteresis and first order reversal curve (FORC) diagrams of specimens from the mudbrick material (SF12E). **(A)** Day Plot [49] following Dunlop (2002) [50]. **(B)** Squareness (Mr/Ms) versus Bc [51]. The different colors in (A)-(B) represent temperatures to which the different specimens were preheated. **(C)** Hysteresis loops of the specimen preheated to 700⁰C. All specimens were saturated at 1.5T. **(D-H)** first order reversal curve (FORC) diagrams of a specimen from the non-heated mud brick material (C) and from specimens preheated to 190⁰C (D), 400⁰C (E), 520⁰C (F), 640⁰C (G) and 700⁰C (H). The diagonal lines across the FORC diagrams are an instrumental artifact and should be ignored. The data show that material heated to temperatures below 520⁰C is dominated by pseudo-single domain (PSD), with contribution of superparamagnetic (SP) and single domain (SD). Increasing the temperature to 520⁰C and higher creates an SD phase with coercivity spectrum that increases with temperature [52].
(EPS)

S5 Fig Representative thermomagnetic curves of the mud brick material (SF12). **(A) N**on-heated material. **(B-D)** material heated to 100⁰C (B) 310⁰C (C) and 700⁰C (D). The curves in (A), (B), and (C) are very similar and show a significant change in the Curie temperature at 600⁰C and 700⁰C. Only the material pre-heated to 700⁰C shows stability up to 700⁰C. The results suggest gradual formation of magnetite and possibly maghemite up to 600⁰C and transformation to hematite between 600⁰C to 700⁰C. All curves were measured using a heating rate of ca. 14⁰C/min.
(EPS)

S6 Fig Viscosity acquisition and destruction curves. **(A)** Results of viscous remanent magnetization (VRM) experiments. We present the mass normalized magnetization of two specimens of crushed unburnt bricks which were placed outdoors in sealed boxes without water (at time 0 on the X-axis). Before the VRM experiment one specimen (SF12E55, marked in blue) was AF demagnetized, after which the two specimens were placed in a Mu-metal shield (<50 μT) within a magnetically shielded room for six days. **(B)** Representative destruction curves (see Extended Methods and S1 Table for description of samples). We present the normalized NRM change of the specimen while being placed in the Mu-metal shield. The initial VRM was recorded by placing the specimens outdoors as in (A) for 9 days. The initial TRM of the remaining six specimens was recorded by heating two of them to each of the temperatures mentioned in the legend (200⁰C, 400⁰C, 600⁰C) in a paleomagnetic oven with a 60μT ambient field.
(EPS)

S7 Fig Color change and disintegrating in water of heated bricks. **(A)** An unoriented specimen taken from the sun-dried bricks collected in the field (SF12E) and 21 specimens taken from the same brick which had been heated in the lab to 100–700⁰C. Notice the gradual change in color which is clearly visible from ~400⁰C and above. **(B)** The same specimens as in (A) after placing them in open paleomagnetic plastic boxes and gently dripping water into the box until the specimens are completely covered in water. The unheated mud brick material and all samples which had been heated to 400⁰C or less disintegrated immediately in the water. Most of them disintegrated into fine grains, with the exception of the sample heated to 400⁰C which broke into coarse grain material. The samples heated to 430⁰C and more were almost unaffected by the water.
(TIF)

S8 Fig The sampled area (SF09). This photo was taken facing west/north-west and shows the opposite of the sampled area shown in Fig 7 in the main text. The locations of SF09A, SF09B and SF09C are marked (SF09C is not visible in this photo). The locations of the segments SF09M and SF09Q are marked by dashed lines.
(TIF)

S9 Fig Representative results of thermal demagnetization which were screened out due to curvature of the Zijderveld [37] diagrams or two-component results. **(A-D)** The results of three specimens from SF09Q which yielded clear two-component results. The component erased at high temperatures alone (marked in green in (B)-(D)) yielded MAD<5 and DANG<5. (A) Displays the calculated directions of the high-temperature components of the two-component specimens and of all specimens of SF09Q which met criteria. Note that the calculated vector of SF09Q15t is pointing above the horizon and that the three vectors are significantly different from each other and from all the rest. B-D display the Zijderveld [37] end-point orthogonal diagrams of the thermal demagnetizations of the three specimens discussed in (A). **(E)** SF09Q49t screened out due to curvature of the Zijderveld diagram.
(EPS)

S10 Fig Representative results of demagnetization experiments on the unoriented brick removed from the wall (SF09N). Zijderveld [37] end-point orthogonal diagrams displaying representative results of demagnetization experiments. The best-fit line is marked in green. The x-axis is rotated to the horizontal direction of the NRM (red circles). Both sub figures represent results from two specimens derived from the same sample (SF09N10) which was roughly in the center of the brick **(A)** Thermal demagnetization**. (B)** AF demagnetization.
(EPS)

S11 Fig Estimated heating temperatures of SF09M. **(A)** Histogram of calculated heating temperatures of all the specimens from the section we cut in the collapsed brick (SF09M). **(B)** Sample SF09M after it was removed from the wall. **(C)** A calculation of the ancient heating temperature in which there are two “knees” in the graph. In this specimen (and one other from SF09M) the first “knee” is below the 0.25 threshold, which could result in an estimation of ~350⁰C for the minimum heating temperature. Therefore, we set the threshold to 0.15 for SF09M only. Since the direction of the magnetization which was erased between the “knees” is the same as that erased above the “knees” (see: (D)) the remaining magnetization between the two “knees” was recorded during the same heating event. **(D)** Zijderveld [37] end-point orthogonal diagram displaying results of the same thermal demagnetization experiment as in (C). The best-fit line is marked in green. **(E)** A calculation of the ancient heating temperature in which there is only one “knee” in the graph.
(TIF)

S12 Fig Estimated heating temperatures of SF09N. **(A)** Histogram of calculated heating temperatures of all the specimens from the section we cut in the unoriented almost intact brick which was removed during the excavation (SF09N). **(B)** Sample SF09N. **(C)** A representative calculation of the ancient heating temperature resulting in a relatively high temperature (580⁰C).
(TIF)

S13 Fig Representative FTIR spectra of SF09M. **(A)** A photo of the collapsed brick (labeled SF09C) after part of its south-western part was removed and its north- eastern part is still in situ. We sampled oriented hand samples from the inner part of the north-eastern section (labeled SF09M). **(B)** SF09M after it was removed. **(C)** Representative FTIR spectra of specimens from SF09M. The location of these specimens (2, 4, 7, 10, 12) can be seen in (B). The dashed black lines mark the area of the Si-O-Al absorption at 518 cm^-1^ (right) and the area of bounded hydroxyls absorption at ~3691cm^-1^ and ~3620cm^-1^ (left). All these peaks are absent, indicating alteration of the clay minerals. The Si-O-Si absorption shifted to 1037cm^-1^ in most spectra but it remained at 1033cm^-1^ in the spectrum of SF09M12 marked in green.
(TIF)

S14 Fig Representative FTIR spectra of SF09Q. **(A)** A photo of an in situ brick which was unearthed in its original orientation within the wall (labeled SF09A). The dashed lines mark the location of the two sections made in SF09A (both labeled SF09Q). The section on the right (specimens SF09Q01-51) was cut for archaeomagnetic experiments after the application of nonmagnetic glue. Since we preferred that the specimens for FTIR not contain this glue, we cut an additional section on the left side of the brick (specimens SF09Q60-68) for FTIR. **(B)** The section made for SF09Q60-68 after the end of the brick had been removed. The location of the different specimens is marked. **(C)** Representative FTIR spectra of specimens from SF09Q. The location of these specimens (61, 63, 65, 67, 68) can be seen in (B). The dashed black lines mark the area of the Si-O-Al absorption at 518cm^-1^ (right) and the area of bounded hydroxyls absorption at ~3691cm^-1^ and ~3620cm^-1^ (left). The Si-O-Al absorption at 518cm^-1^ is clearly visible and the Si-O-Si absorption is at 1033cm^-1^ in all spectra. Although the peaks of bounded hydroxyls absorption at ~3691cm^-1^ and ~3620cm^-1^ are not very clear, these spectra cannot be used as an indication of heat-altered clay.
(TIF)

S1 Table Experiments carried out on the unheated clay in order to study the viscous remanent magnetization (VRM) acquisition and destruction curves. The different steps which were carried out are described in detail in the “Extended methods” section in S1 Text.
(PDF)

S2 Table Archaeomagnetic direction results from thermal demagnetization experiments carried out on SF09M (all specimens met the MAD and DANG criteria). For every specimen the table shows the mean direction (declination and inclination), the temperature range (Tmin and Tmax), the number of demagnetization steps which were chosen from Tmin to Tmax (n), the (*non-anchored*) Maximum Angular Deviation (MAD) parameter to quantify the scatter of the points on the Zijderveld plots [38] and the Deviation Angle (DANG) to quantify convergence toward the origin [39].
(PDF)

S3 Table Archaeomagnetic direction results from thermal demagnetization experiments carried out on SF09Q (only specimens that yielded univectorial results and met the MAD and DANG criteria). For every specimen the table shows the mean direction (declination and inclination), the temperature range (Tmin and Tmax), the number of demagnetization steps which were chosen from Tmin to Tmax (n), the (*non-anchored*) Maximum Angular Deviation (MAD) parameter to quantify the scatter of the points on the Zijderveld plots [38] and the Deviation Angle (DANG) to quantify convergence toward the origin [39]. The results of three specimens from SF09Q which yielded clear two-component results (S9B-S9D Fig) are not included in the table.
(PDF)

S4 Table Archaeomagnetic direction results from the wall and the collapsed material around it (SF09) according to sample. For every sample the table shows the mean direction (declination and inclination), the number of specimens used for Fisher statistics (N) out of all specimens which met the MAD and DANG criteria (N_0_), the Fisher precision parameter (k) and the 95% confidence cone angle (α_95_). The k and the α_95_ were calculated using Fisher statistics [40].
(PDF)

S5 Table Archaeomagnetic direction results from thermal demagnetization experiments carried out on SF09N (all specimens met the MAD and DANG criteria). For every specimen the table shows the mean direction (declination and inclination), the temperature range (Tmin and Tmax), the number of demagnetization steps which were chosen from Tmin to Tmax (n), the (*non-anchored*) Maximum Angular Deviation (MAD) parameter to quantify the scatter of the points on the Zijderveld plots [38] and the Deviation Angle (DANG) to quantify convergence toward the origin [39].
(PDF)

S1 Text Extended methods and Extended results. (PDF)

S1 File MagIC format data file containing the demagnetization measurements of the experimentally magnetized miniature “mudbricks” (SF12E). For instructions regarding MagIC files see: https://pmagpy.github.io/PmagPy-docs/intro.html.
(TXT)

S2 File MagIC format data file containing the demagnetization measurements of specimens from the wall and the collapsed material around it (SF09). For instructions regarding MagIC files see: https://pmagpy.github.io/PmagPy-docs/intro.html.
(TXT)
