# Supplementary figures and images for: Applying thermal demagnetization to archaeological materials: A tool for detecting burnt clay and estimating its firing temperature
Source: PLoS One. 2023 Oct 9;18(10):e0289424. doi: 10.1371/journal.pone.0289424 (PMC10561874; doi:10.1371/journal.pone.0289424)

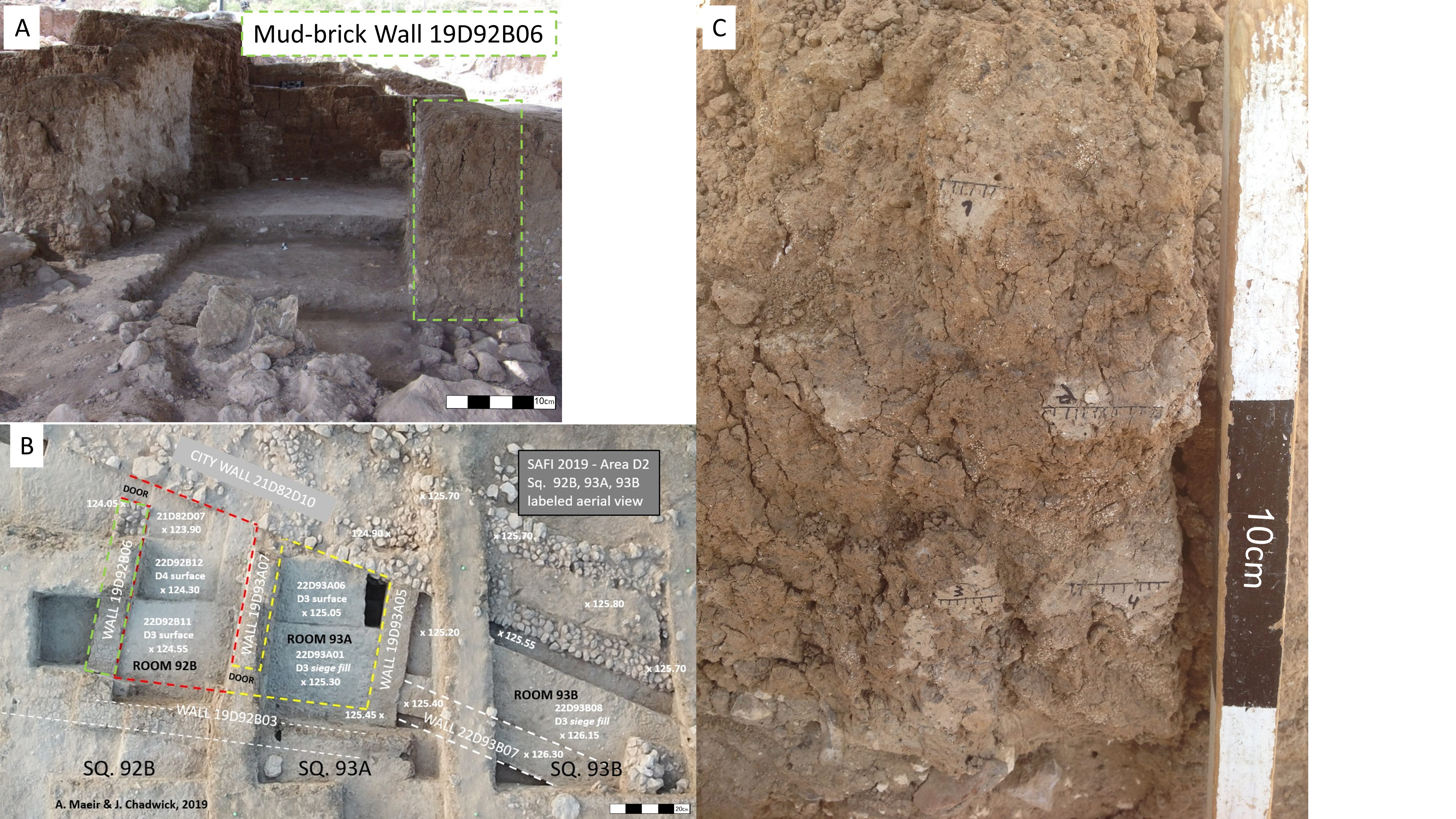

Supplement: S1 Fig — (A) A profile view of Wall 19D92B06 (view looking south), with east side clean but west side still abutted by fill. (B) An aerial photo of Squares 92B, 93A and 93B in Area D2. Wall 19D92B06 is marked by a green dashed rectangle in both (A) and (B). (C) Oriented samples of in-situ mud bricks (SF12A01-04; close up view, looking south). (TIF) [file pone.0289424.s001.tif]

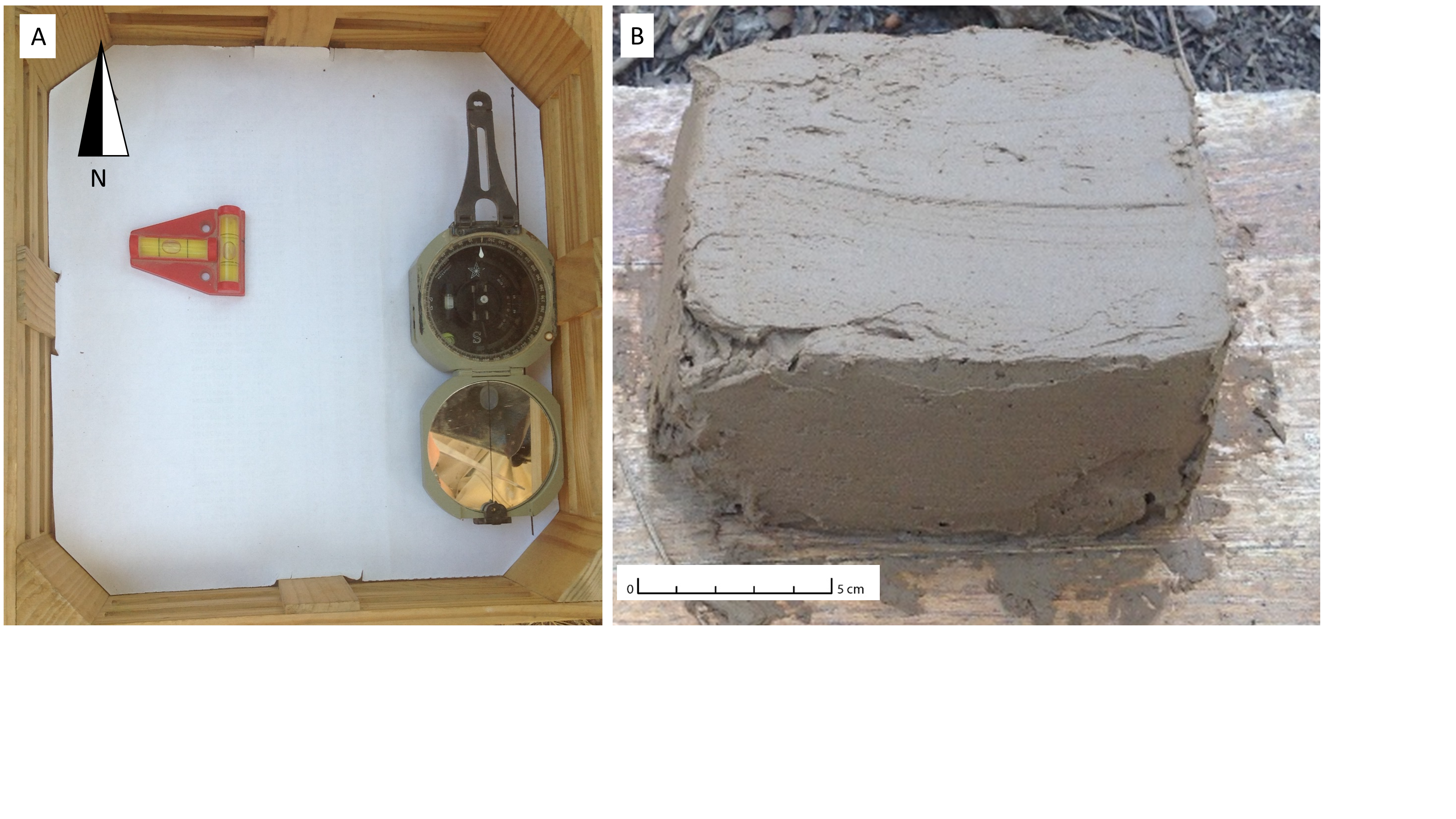

Supplement: S3 Fig — (A) The wooden tray placed horizontally in the field away from modern magnetic disturbances and oriented facing to the magnetic north using a Brunton compass. (B) An experimental mud brick made from the same mixture of crushed material and water as the miniature “bricks” in order to make sure that the brick maintains its shape without a cast. (TIF) [file pone.0289424.s003.tif]

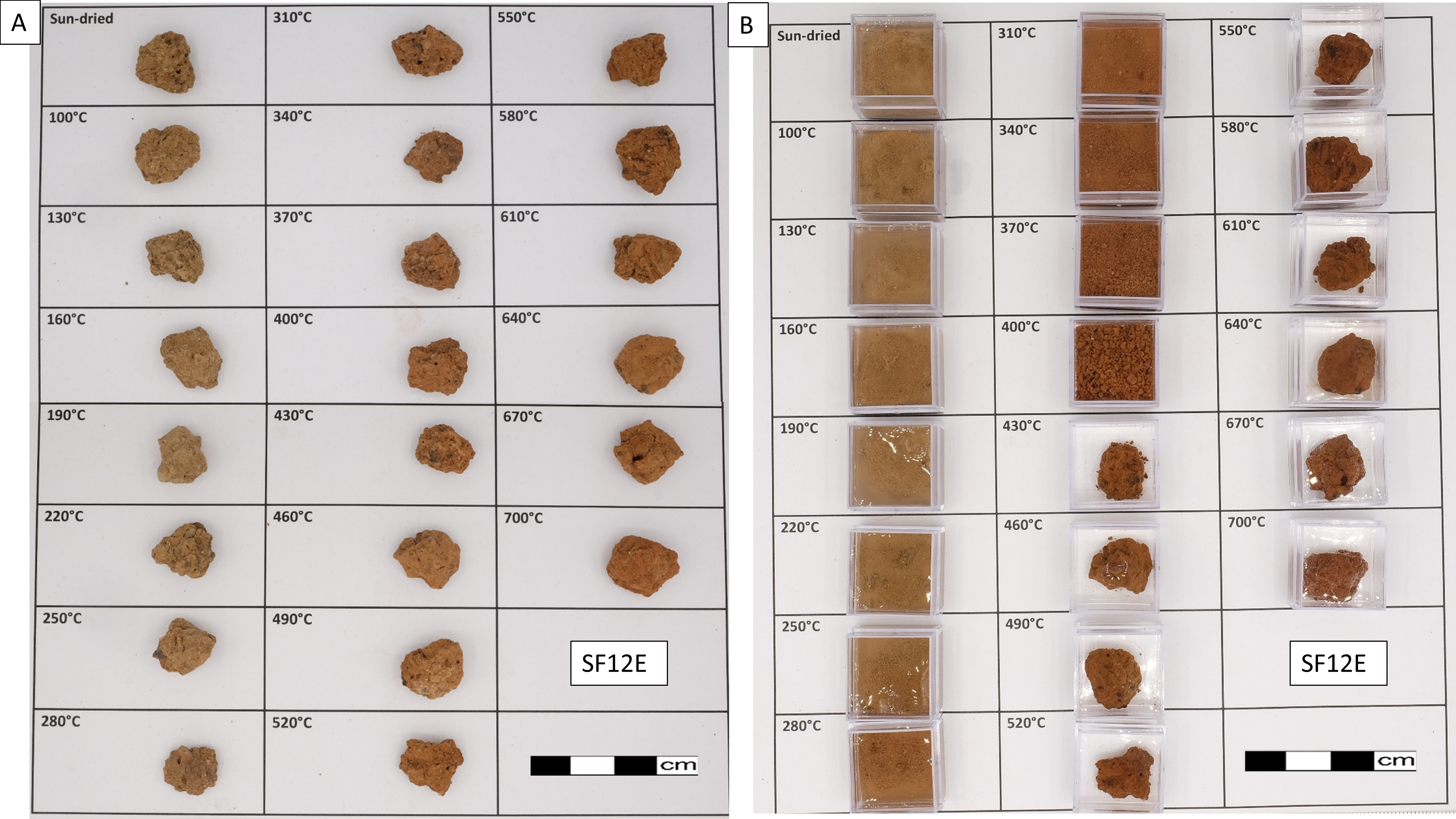

Supplement: S7 Fig — (A) An unoriented specimen taken from the sun-dried bricks collected in the field (SF12E) and 21 specimens taken from the same brick which had been heated in the lab to 100–700⁰C. Notice the gradual change in color which is clearly visible from ~400⁰C and above. (B) The same specimens as in (A) after placing them in open paleomagnetic plastic boxes and gently dripping water into the box until the specimens are completely covered in water. The unheated mud brick material and all samples which had been heated to 400⁰C or less disintegrated immediately in the water. Most of them disintegrated into fine grains, with the exception of the sample heated to 400⁰C which broke into coarse grain material. The samples heated to 430⁰C and more were almost unaffected by the water. (TIF) [file pone.0289424.s007.tif]

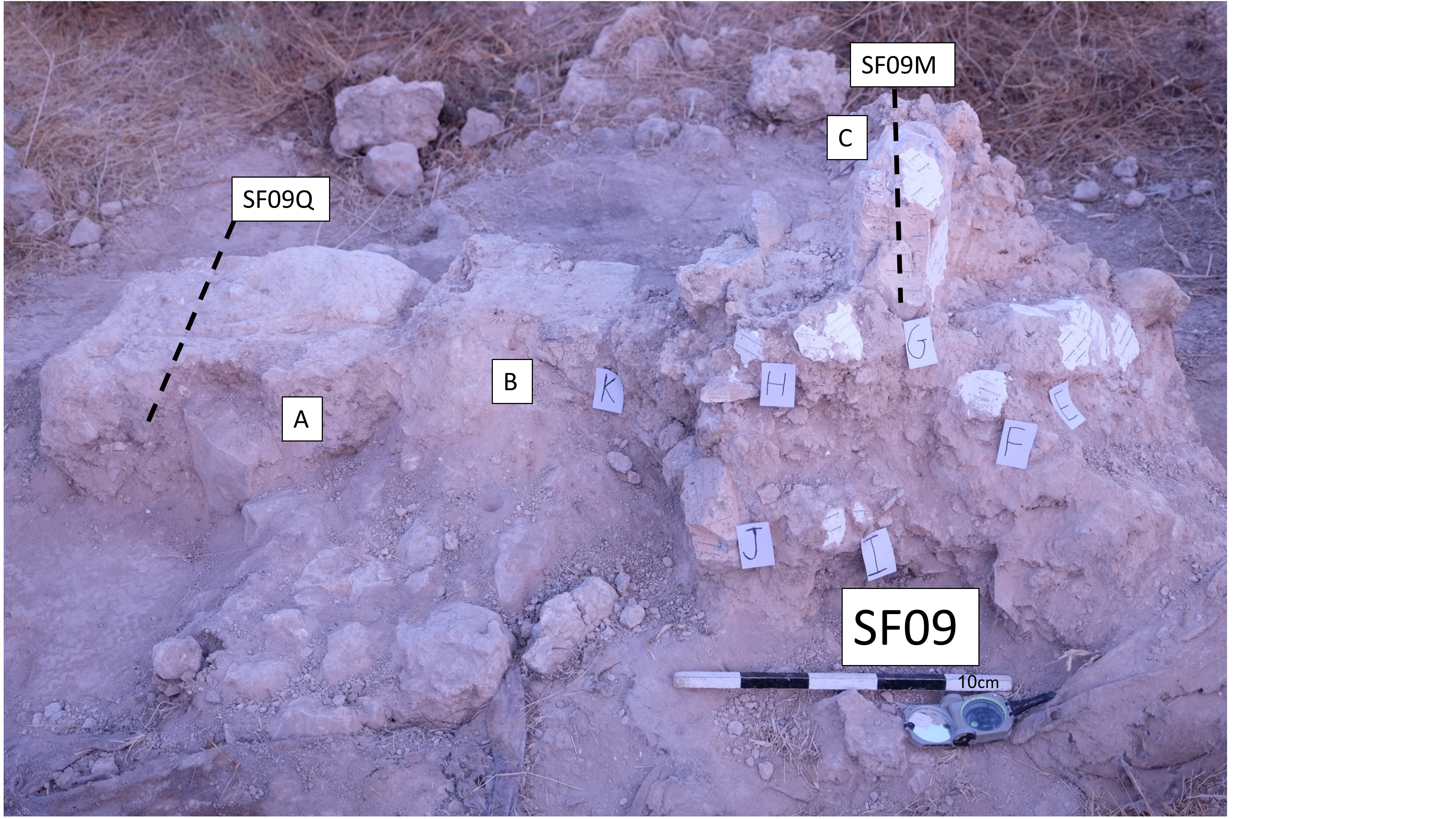

Supplement: S8 Fig — This photo was taken facing west/north-west and shows the opposite of the sampled area shown in Fig 7 in the main text. The locations of SF09A, SF09B and SF09C are marked (SF09C is not visible in this photo). The locations of the segments SF09M and SF09Q are marked by dashed lines. (TIF) [file pone.0289424.s008.tif]

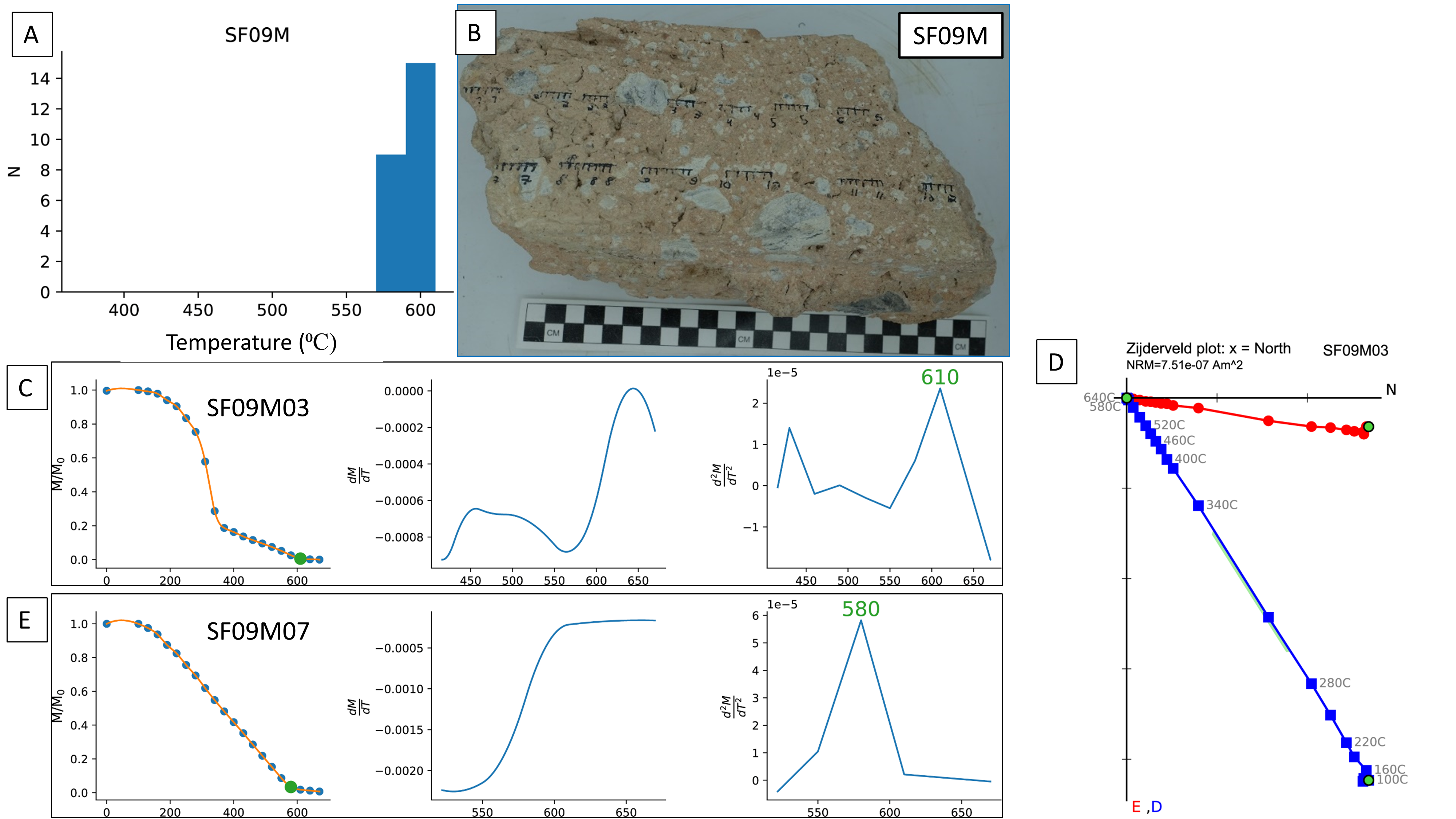

Supplement: S11 Fig — (A) Histogram of calculated heating temperatures of all the specimens from the section we cut in the collapsed brick (SF09M). (B) Sample SF09M after it was removed from the wall. (C) A calculation of the ancient heating temperature in which there are two “knees” in the graph. In this specimen (and one other from SF09M) the first “knee” is below the 0.25 threshold, which could result in an estimation of ~350⁰C for the minimum heating temperature. Therefore, we set the threshold to 0.15 for SF09M only. Since the direction of the magnetization which was erased between the “knees” is the same as that erased above the “knees” (see: (D)) the remaining magnetization between the two “knees” was recorded during the same heating event. (D) Zijderveld [37] end-point orthogonal diagram displaying results of the same thermal demagnetization experiment as in (C). The best-fit line is marked in green. (E) A calculation of the ancient heating temperature in which there is only one “knee” in the graph. (TIF) [file pone.0289424.s011.tif]

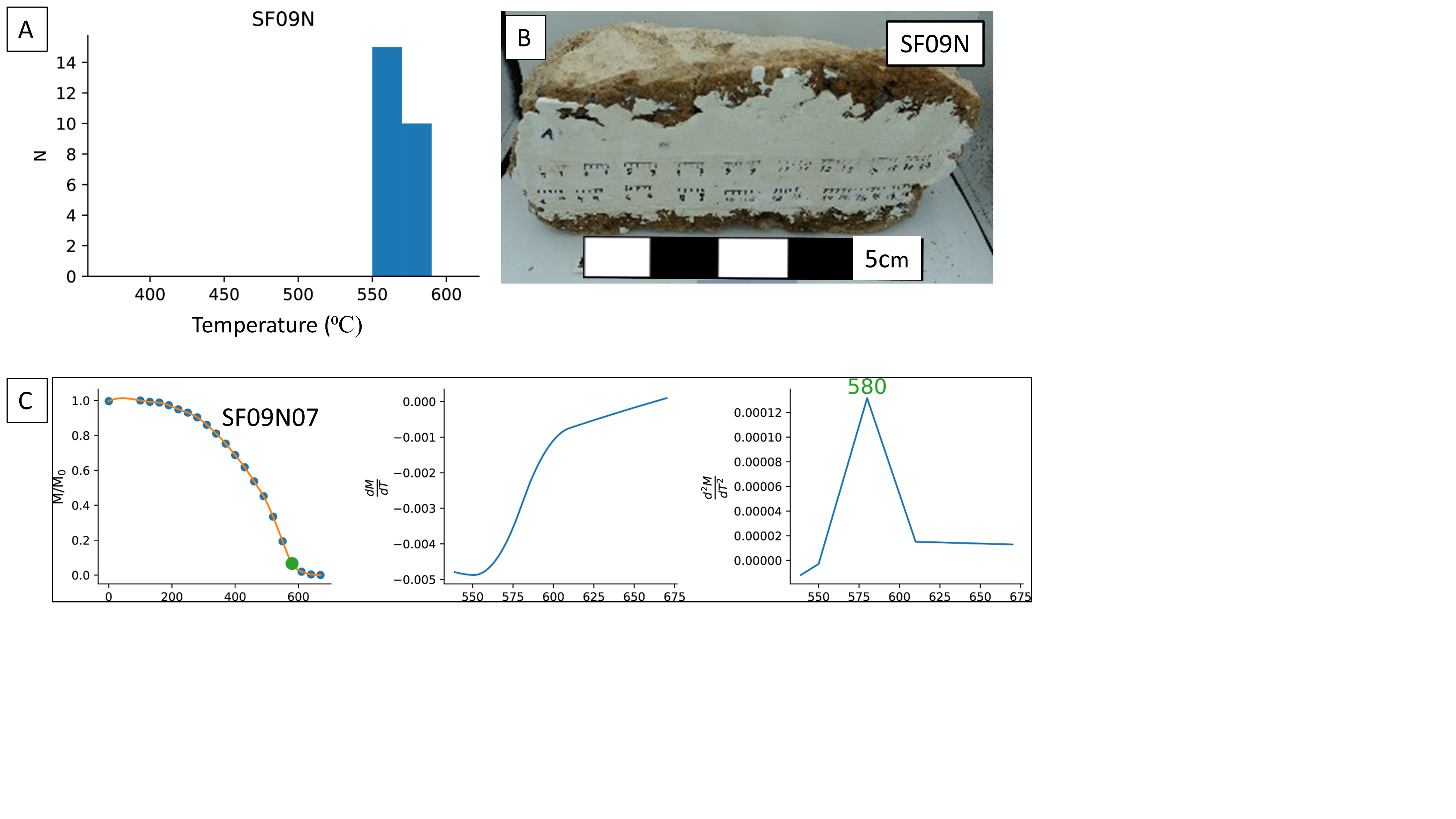

Supplement: S12 Fig — (A) Histogram of calculated heating temperatures of all the specimens from the section we cut in the unoriented almost intact brick which was removed during the excavation (SF09N). (B) Sample SF09N. (C) A representative calculation of the ancient heating temperature resulting in a relatively high temperature (580⁰C). (TIF) [file pone.0289424.s012.tif]

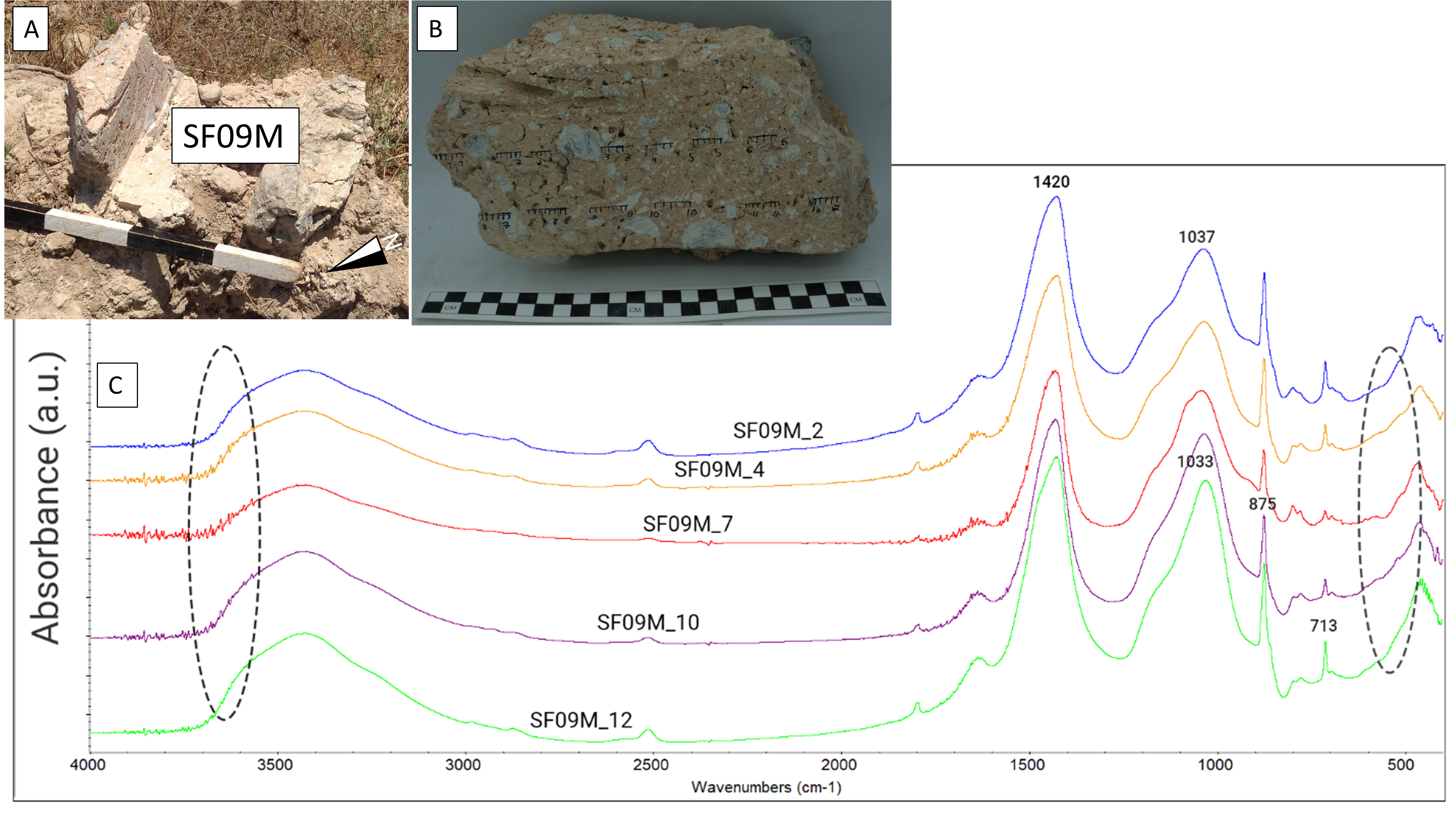

Supplement: S13 Fig — (A) A photo of the collapsed brick (labeled SF09C) after part of its south-western part was removed and its north- eastern part is still in situ. We sampled oriented hand samples from the inner part of the north-eastern section (labeled SF09M). (B) SF09M after it was removed. (C) Representative FTIR spectra of specimens from SF09M. The location of these specimens (2, 4, 7, 10, 12) can be seen in (B). The dashed black lines mark the area of the Si-O-Al absorption at 518 cm-1 (right) and the area of bounded hydroxyls absorption at ~3691cm-1 and ~3620cm-1 (left). All these peaks are absent, indicating alteration of the clay minerals. The Si-O-Si absorption shifted to 1037cm-1 in most spectra but it remained at 1033cm-1 in the spectrum of SF09M12 marked in green. (TIF) [file pone.0289424.s013.tif]

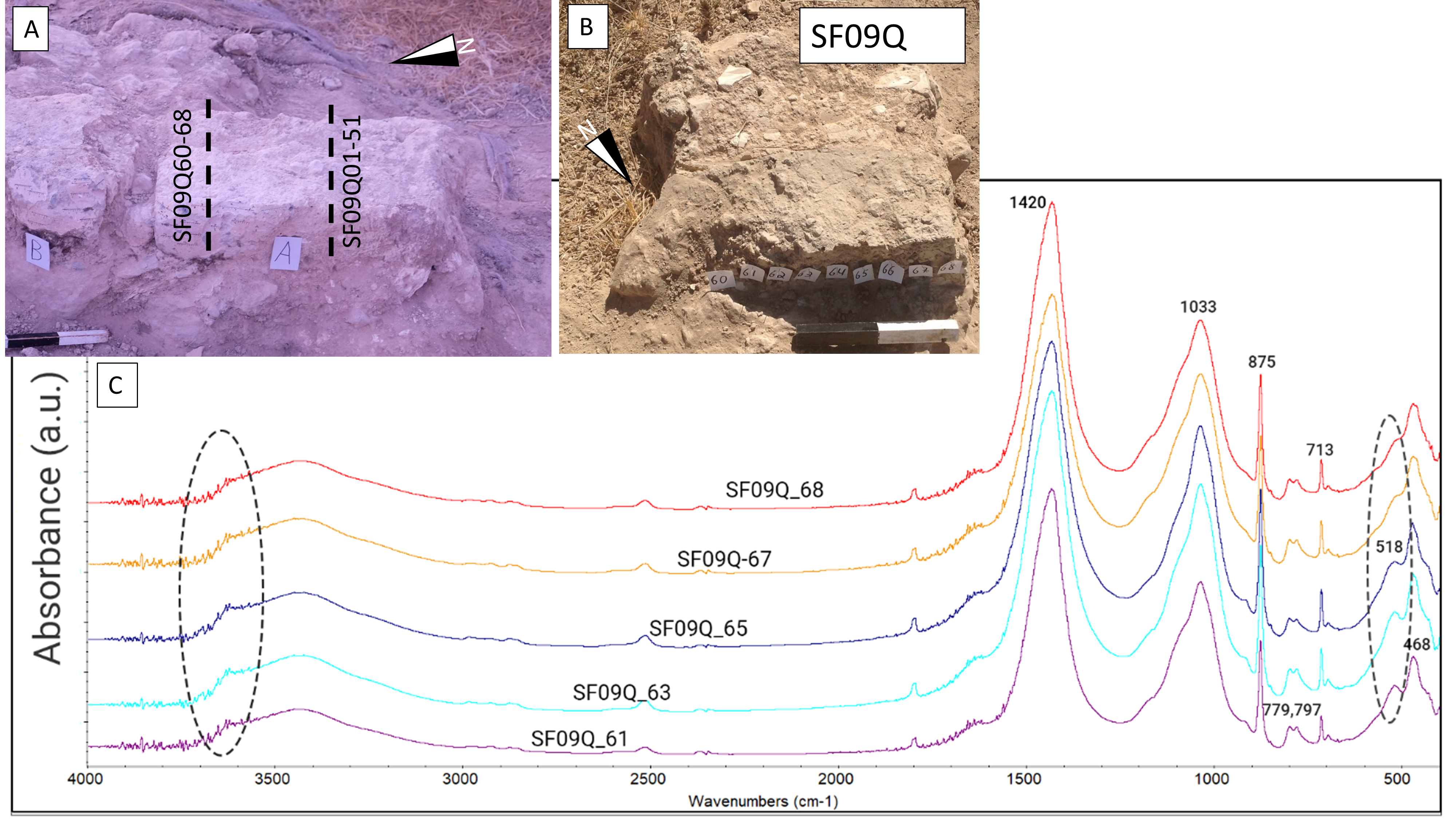

Supplement: S14 Fig — (A) A photo of an in situ brick which was unearthed in its original orientation within the wall (labeled SF09A). The dashed lines mark the location of the two sections made in SF09A (both labeled SF09Q). The section on the right (specimens SF09Q01-51) was cut for archaeomagnetic experiments after the application of nonmagnetic glue. Since we preferred that the specimens for FTIR not contain this glue, we cut an additional section on the left side of the brick (specimens SF09Q60-68) for FTIR. (B) The section made for SF09Q60-68 after the end of the brick had been removed. The location of the different specimens is marked. (C) Representative FTIR spectra of specimens from SF09Q. The location of these specimens (61, 63, 65, 67, 68) can be seen in (B). The dashed black lines mark the area of the Si-O-Al absorption at 518cm-1 (right) and the area of bounded hydroxyls absorption at ~3691cm-1 and ~3620cm-1 (left). The Si-O-Al absorption at 518cm-1 is clearly visible and the Si-O-Si absorption is at 1033cm-1 in all spectra. Although the peaks of bounded hydroxyls absorption at ~3691cm-1 and ~3620cm-1 are not very clear, these spectra cannot be used as an indication of heat-altered clay. (TIF) [file pone.0289424.s014.tif]
