# Supplementary material for: Applying thermal demagnetization to archaeological materials: A tool for detecting burnt clay and estimating its firing temperature
Source: PLoS One. 2023 Oct 9;18(10):e0289424. doi: 10.1371/journal.pone.0289424 (PMC10561874; doi:10.1371/journal.pone.0289424)
Supplement: S1 Table — The different steps which were carried out are described in detail in the “Extended methods” section in S1 Text. (PDF) [file pone.0289424.s015.pdf]

Specimen names	No.	Crushed?	Container	Step I	Step II	Step III	Step IV
SF12E55	1	yes	Plastic box	AF demag.	VRM_0	VRM	-
SF12E56	1	yes	Plastic box	AF demag.	VRM_0	VRM	SIRM
SF12E57	1	yes	Plastic box	AF demag.	VRM_0	VRM	VRM_0
SF12E58	1	yes	Plastic box	VRM_0	VRM	-	-
SF12E59	1	yes	Plastic box	VRM_0	VRM	SIRM	-
SF12E60	1	yes	Plastic box	VRM_0	VRM	VRM_0	-
SF12E85-86, 89-90, 93-94	6	yes	Crucibles	In-field heating	VRM	-	-
SF12E87-88, 91-92, 95-96	6	yes	Crucibles	In-field heating	VRM_0	-	-
