# Supplementary material for: Applying thermal demagnetization to archaeological materials: A tool for detecting burnt clay and estimating its firing temperature
Source: PLoS One. 2023 Oct 9;18(10):e0289424. doi: 10.1371/journal.pone.0289424 (PMC10561874; doi:10.1371/journal.pone.0289424)
Supplement: S2 Table — For every specimen the table shows the mean direction (declination and inclination), the temperature range (Tmin and Tmax), the number of demagnetization steps which were chosen from Tmin to Tmax (n), the (non-anchored) Maximum Angular Deviation (MAD) parameter to quantify the scatter of the points on the Zijderveld plots [38] and the Deviation Angle (DANG) to quantify convergence toward the origin [39]. (PDF) [file pone.0289424.s016.pdf]

Specimen	Dec	Inc	Tmin	Tmax	n	MAD	DANG
SF09M01t	1.5	62.3	100	670	20	2.4	1
SF09M01u	8.3	65.1	100	670	20	1.2	0.5
SF09M02t	3.8	61.5	100	670	20	1	1
SF09M02u	11	59.9	100	670	20	1.3	0.8
SF09M03t	7.9	57.4	100	670	20	0.8	0.4
SF09M03u	9.1	59.7	100	670	20	1	0.4
SF09M04t	3.9	62.3	100	670	20	1.2	0.2
SF09M04u	359.6	59	100	670	20	0.5	0.1
SF09M05t	8.8	59.4	100	670	20	0.8	0.4
SF09M05u	11.5	59.4	100	670	20	0.5	0.4
SF09M06t	9.5	60	100	670	20	0.3	0.2
SF09M06u	12.6	61.6	100	670	20	0.7	0.3
SF09M07t	359.2	59.6	100	670	20	0.9	0.1
SF09M07u	4.6	54.9	100	670	20	1	0.2
SF09M08t	5.9	62.5	100	670	20	0.3	0.2
SF09M08u	6.1	59.5	100	670	20	0.5	0.2
SF09M09t	6.7	61	100	670	20	0.6	0.2
SF09M09u	4.8	59.7	100	670	20	0.6	0.4
SF09M10t	9.7	59.2	100	670	20	0.9	0.7
SF09M10u	5.7	59.6	100	670	20	1.1	0.9
SF09M11t	6.2	63.1	100	670	20	1.2	1.1
SF09M11u	1.8	62.1	100	670	20	1.4	1.1
SF09M12t	7.1	61.4	100	670	20	0.6	0.4
SF09M12u	17.5	58.9	100	670	20	1.3	0.2
