# Supplementary material for: Applying thermal demagnetization to archaeological materials: A tool for detecting burnt clay and estimating its firing temperature
Source: PLoS One. 2023 Oct 9;18(10):e0289424. doi: 10.1371/journal.pone.0289424 (PMC10561874; doi:10.1371/journal.pone.0289424)
Supplement: S3 Table — For every specimen the table shows the mean direction (declination and inclination), the temperature range (Tmin and Tmax), the number of demagnetization steps which were chosen from Tmin to Tmax (n), the (non-anchored) Maximum Angular Deviation (MAD) parameter to quantify the scatter of the points on the Zijderveld plots [38] and the Deviation Angle (DANG) to quantify convergence toward the origin [39]. The results of three specimens from SF09Q which yielded clear two-component results (S9B-S9D Fig) are not included in the table. (PDF) [file pone.0289424.s017.pdf]

Specimen	Dec	Inc	Tmin	Tmax	n	MAD	DANG
SF09Q01t	3.8	57.4	100	670	20	0.7	0.3
SF09Q02t	15.4	49.1	100	670	20	1.2	0.7
SF09Q03t	12.5	51.4	100	670	20	1.5	0.8
SF09Q04t	9.4	55.2	100	670	20	1.4	1.3
SF09Q05t	4.9	62.5	100	670	20	0.6	0.8
SF09Q06t	359.4	54.2	100	670	20	0.6	0.6
SF09Q07t	1.3	53.7	100	670	20	1	1.3
SF09Q08t	13.5	56	100	670	20	1.4	1.2
SF09Q10t	7.2	61.1	100	670	20	0.7	0.5
SF09Q11t	6.9	60.7	100	670	20	1	1
SF09Q12t	12.2	55.8	100	670	20	1.5	0.9
SF09Q13t	359.2	59.9	100	670	20	0.9	0.6
SF09Q14t	4.6	59.6	100	670	20	0.5	0.3
SF09Q16t	7.9	60.1	100	670	20	1	1.1
SF09Q17t	14	59.6	100	670	20	1	1
SF09Q18t	10.3	57.4	100	670	20	0.8	1
SF09Q19t	360	61	100	670	20	0.9	1.1
SF09Q20t	4.2	59.5	100	670	20	0.7	1.2
SF09Q21t	5.8	58.8	100	670	20	1.1	0.8
SF09Q22t	359.7	58.4	100	670	20	1.5	0.6
SF09Q23t	13.5	53.3	100	670	20	1.3	1.2
SF09Q24t	1.7	52.8	100	670	20	0.7	0.7
SF09Q25t	357.8	55.3	100	670	20	1.6	1.4
SF09Q26t	7.7	56.1	100	670	20	0.9	0.3
SF09Q27t	3.2	59.3	100	670	20	1.3	1.3
SF09Q28t	6.8	56.8	100	670	20	0.9	1.1
SF09Q29t	18.3	48.7	100	670	20	3.5	1.6
SF09Q30t	21.4	61.3	100	670	20	1.6	1
SF09Q31t	50.8	48.8	100	670	20	3.8	4
SF09Q32t	2.1	59.2	100	670	20	1.5	1.9
SF09Q34t	357.5	50.6	100	670	20	1.5	1.4
SF09Q35t	23.7	56.3	100	670	20	1.6	0.8
SF09Q36t	26.7	50.7	100	670	20	3.2	5
SF09Q37t	9.3	52.4	100	670	20	1.4	2
SF09Q38t	359.3	52.7	100	670	20	1.5	0.9
SF09Q40t	0.3	54.7	100	670	20	2.6	2.2
SF09Q41t	9.4	48.6	100	670	20	1.5	0.8
SF09Q42t	5	51.3	100	670	20	3.4	2.1
SF09Q43t	3.4	51.4	100	670	20	1.1	1
SF09Q45t	358.6	33.2	100	670	20	3.1	3.1
SF09Q46t	30.7	31.1	0	580	18	2.3	2.9
SF09Q48t	7.2	49.9	100	670	20	3.2	2.9
SF09Q50t	3.3	31.7	100	670	20	4.9	4.8
SF09Q51t	3.6	41.1	100	670	20	2.5	0.6
