# Supplementary material for: Applying thermal demagnetization to archaeological materials: A tool for detecting burnt clay and estimating its firing temperature
Source: PLoS One. 2023 Oct 9;18(10):e0289424. doi: 10.1371/journal.pone.0289424 (PMC10561874; doi:10.1371/journal.pone.0289424)
Supplement: S4 Table — For every sample the table shows the mean direction (declination and inclination), the number of specimens used for Fisher statistics (N) out of all specimens which met the MAD and DANG criteria (N0), the Fisher precision parameter (k) and the 95% confidence cone angle (α95). The k and the α95 were calculated using Fisher statistics [40]. (PDF) [file pone.0289424.s018.pdf]

Sample	Dec	Inc	N/N_0	k	α_{95}
SF09A	13	53.8	10/10	239	3.1
SF09B	16.2	62.3	8/8	327	3.1
SF09C	17.7	60.7	6/6	531	2.9
SF09D	3.5	62.5	6/6	211	4.6
SF09E	8.5	63.8	9/9	1098	1.6
SF09F	9.6	64.6	6/6	2023	1.5
SF09G	10.1	66.3	5/6	732	2.8
SF09H	344.2	65.9	6/6	237	4.4
SF09I	353.6	68.3	6/6	120	6.1
SF09J	1.1	60.8	6/6	880	2.3
SF09K	10.5	61.5	6/6	155	5.4
SF09M	4.9	60.8	48/48	679	0.8
SF09Q	6.7	54.7	91/91	85	1.6

References
