# Supplementary material for: Applying thermal demagnetization to archaeological materials: A tool for detecting burnt clay and estimating its firing temperature
Source: PLoS One. 2023 Oct 9;18(10):e0289424. doi: 10.1371/journal.pone.0289424 (PMC10561874; doi:10.1371/journal.pone.0289424)
Supplement: S5 Table — For every specimen the table shows the mean direction (declination and inclination), the temperature range (Tmin and Tmax), the number of demagnetization steps which were chosen from Tmin to Tmax (n), the (non-anchored) Maximum Angular Deviation (MAD) parameter to quantify the scatter of the points on the Zijderveld plots [38] and the Deviation Angle (DANG) to quantify convergence toward the origin [39]. (PDF) [file pone.0289424.s019.pdf]

Specimen	Dec	Inc	Tmin	Tmax	n	MAD	DANG
SF09N01t	8	61.9	100	670	20	0.5	0.2
SF09N02t	6.5	59	100	670	20	0.7	0.3
SF09N03t	6.8	61.9	100	670	20	0.9	0.3
SF09N04t	7.4	60.5	100	670	20	1.1	0.3
SF09N04u	7.6	59.7	100	670	20	1.1	0.2
SF09N05t	10.6	59.8	100	670	20	0.6	0.3
SF09N05u	9.2	59.1	100	670	20	0.5	0.3
SF09N06t	6.7	62	100	670	20	0.6	0.2
SF09N06u	5.7	62.3	100	670	20	1	0.3
SF09N07t	10.5	59.9	100	670	20	1.1	0.5
SF09N07u	5.5	58.9	100	670	20	1.6	0.1
SF09N08t	11.2	54.4	100	670	20	0.6	0.2
SF09N08u	11.2	59	100	670	20	0.9	0.4
SF09N09t	12.4	59.2	100	670	20	1.2	0.3
SF09N10t	7.4	60.5	100	670	20	0.9	0.3
SF09N11t	10.7	60	100	670	20	0.5	0.2
SF09N11u	8	60.2	100	670	20	1	0.1
SF09N12t	12.2	59.5	100	670	20	1.3	0.3
SF09N12u	12.6	61.1	100	670	20	0.5	0.1
SF09N13t	13.1	61.6	100	670	20	0.9	0.2
SF09N13u	10.2	60.5	100	670	20	0.5	0.1
SF09N14t	12.9	60.6	100	670	20	1	0.3
SF09N14u	14.8	60.5	100	670	20	1.2	0.2
SF09N15t	11.2	59.2	100	670	20	0.9	0.2
SF09N16t	8.7	59.2	100	670	20	1	0.4
