# Supplementary material for: Applying thermal demagnetization to archaeological materials: A tool for detecting burnt clay and estimating its firing temperature
Source: PLoS One. 2023 Oct 9;18(10):e0289424. doi: 10.1371/journal.pone.0289424 (PMC10561874; doi:10.1371/journal.pone.0289424)
Supplement: S1 Text — (PDF) [file pone.0289424.s020.pdf]

Supplementary Text for:

Applying thermal demagnetization to archaeological materials: A tool for detecting burnt clay and estimating its firing temperature

Yoav Vaknin[1,2*], Ron Shaar[2], Oded Lipschits[1], Adi Eliyahu Behar[3], Aren M. Maeir[4], Erez Ben-Yosef[1]

[1] Institute of Archaeology, Tel Aviv University; Tel Aviv, Israel.

[2] Institute of Earth Sciences, The Hebrew University of Jerusalem; Jerusalem, Israel.

[3] The Department of Land of Israel Studies and Archaeology and the Department of Chemical Sciences, Ariel University; Ariel, Israel

[4] The Institute of Archaeology, The Martin (Szusz) Department of Land of Israel Studies and Archaeology, Bar-Ilan University; Ramat Gan, Israel.

* Corresponding author

E-mail: yoavvaknin@mail.tau.ac.il (YV)

Extended Methods

The identification of the mud bricks sampled in Area D2 (SF12) as unburnt brick material

We chose to develop the new method using genuine mud brick material in order to develop and test our method on materials as close as possible to those we examined in the case study and those on which we wish to implement it in the future. Therefore, we recreated the ancient procedure of mud brick construction, which often involved recycling mud bricks that had gone out of use [1-3]. However, due to our choice and the results of this research, it is impossible to rule out the possibility that the apparently unheated mud bricks were heated to temperatures below ~190ºC after they were dried and before they were placed in the wall. This possibility, however, seems very unlikely, since there is no practical advantage in such a practice. Even if it were the case, the crushing and mixing of the mud brick material before making our miniature "mud bricks" resets any magnetization recorded in the material, and thus it does not affect our results and conclusions. The only effect of possibly non-detected pre-heating of the mud bricks is on the interpretation of the rock magnetic experiments (Fig 4, S4-S6 Figs).

We assumed in the field that these bricks were unburnt since no signs of fire were unearthed in their vicinity and the entire area was deliberately covered over and buried with sediments in antiquity, before any destruction occurred. The color of the bricks was brown (S1A, C Fig). They tended to crumble and immediately disintegrated when placed in water (S5 Fig), suggesting that these bricks had not been heated to 400ºC or more. FTIR analysis showed no signs of heated clays (Fig 6A in the main text), which corroborates that these bricks have not been heated to 500ºC or more. The change in susceptibility after heating to ~200-250ºC (Fig 4A) suggests that the minerals had not been heated to this temperature; otherwise the susceptibility would have remained stable.

In the field, we hardened small parts of the outer surface of in-situ mud bricks using non-magnetic potassium silicate glue (Kasil). We then polished flat surfaces on the outer part of these bricks and sampled them as oriented "hand samples" (S1C Fig). Four oriented specimens from these samples (SF12A) were demagnetized using AF demagnetization, at progressively elevated peak field in 4mT steps up to 20mT, 5mT steps up to 40mT, 10mT steps up to 70mT and 15mT steps up to 100mT. This preliminary experiment was carried out in order to test whether these bricks had been heated. Two out of four of these specimens failed criteria (MAD>5º and/or DANG>5º) and all yielded directions largely scattered around the east/south-east with a small positive inclination (S2 Fig), which is completely different from the expected geomagnetic field direction. This indicates that these weak magnetic signals were recorded in different orientations from those in which these bricks were unearthed and rules out the possibility that they were burnt in-situ. The low coercivity of these specimens is demonstrated by the entire removal of the magnetic moment at 20-40mT. Comparing these results (S2 Fig) with those of the sun-dried bricks we prepared under controlled conditions and the thermally magnetized bricks made from the same material (Fig 2 in the main text) shows that the weak and often non-unified magnetic signal of SF12A was most likely recorded when these bricks were sun-dried. It is of course possible that some of the mud used to manufacture these bricks had been heated

in the past (for instance, bricks burnt in destruction events, mud ovens, etc.) and found its way into the mud composition. In any case, the influence of small pieces of burnt material and/or the ancient heating of these bricks to less than 190ºC on the present research is negligible, since we erased any magnetic signal when we crushed and mixed this material.

The Python script for calculating the ancient heating temperature

We uploaded the Python script which we wrote and tested for calculating the ancient temperature to the supplementary material. This script takes as input a MagIC formatted measurement file (measurements.txt) and calculates the ancient temperature from any thermal demagnetization data in the file.

The script uses PmagPy functions available at:

https://github.com/PmagPy/PmagPy

For information and installation of the PmagPy software package:

https://earthref.org/PmagPy/cookbook/

https://pmagpy.github.io/

Acquisition and destruction of viscous remanent magnetization (VRM) in "mud bricks" (see S1 Table)

In the following we describe an experimental procedure designed to explore the effect of VRM on the sun-dried mud bricks as well as on the heated mud. The steps of the various experiments are summarized in S1 Table and are described as follows:

Initial VRM_0 (VRM destruction): In order to distinguish between DhRM and viscous remanent magnetization (VRM) we prepared six specimens (SF12E55-60) from crushed unburnt mudbrick material. We placed this material in sealed paleomagnetic plastic boxes in order to prevent exposure to humidity. In order to remove any initial magnetization, three specimens (SF1255-57) underwent AF demagnetization (in the steps mentioned above) and then all six specimens were placed in a Mu-metal shield (B < 50 nT) for six days.

In-field heating: We thermally magnetized 12 additional specimens (SF12E85-96) in an ambient oven field of 60μT in a "south" and horizontal direction, four specimens at every one of these temperatures: 200ºC, 400ºC, 600ºC, and measured their TRM.

VRM (acquisition): After measuring their magnetization, we placed the six specimens which had undergone initial VRM_0 (SF12E55-60) and six specimens which recorded TRM (SF12E85-86, 89-90, 93-94) outdoors, in the same location where SF12E01-51 had been sun-dried, and their magnetization was measured regularly in order to follow the VRM acquisition. Every time these specimens were transferred to the lab to be measured for VRM and back to the outdoor location they were carried in a Mu-metal shield.

VRM_0 (VRM destruction): In order to create destruction curves we placed two of the six specimens which recorded VRM (SF12E57, 60) and six specimens which recorded TRM (two which were heated to each temperature (200ºC, 400ºC, 600ºC): SF12E87-88, 91-92,

95-96) in the Mu-metal shield inside the magnetically shielded room and regularly measured their magnetization.

SIRM: In order to compare the VRM to the saturation isothermal remanent magnetization (SIRM) we magnetized two of the VRM specimens (SF12E56, 59) to saturation in a 1.4T field and measured this magnetization within 2 minutes. The VRM normalized to the SIRM helps distinguish between VRM and DhRM (Fig 4D in the main text).

Extended Results

The importance of a unified magnetic signal for estimating the firing temperature

Ignoring the MAD and DANG criteria might result in unreliable temperature estimations. For instance, the temperature estimation of SF09Q09t would have been 550ºC. The magnetic moment of this specimen is clearly comprised of two different components (S7B Fig). The component erased in the 100-430ºC steps was roughly in the direction of all other samples. However, the second component erased during the 460-550ºC steps (marked in green in Fig 9D) is a horizontal vector pointing roughly to the north-east (Declination=48.5 Inclination=3.5). Even though this second component meets MAD and DANG criteria if one ignores the lower temperature steps (MAD=1.7, DANG=0.9) it should not be used for reconstructing the heating temperature. Perhaps this magnetic signal is the result of a piece of burnt mud, stone, pottery or any other magnetic material which had been mixed by chance into the mud composition of SF09Q. If this was the case, when the area of this specimen (SF09Q09t) within the brick (SF09Q) was heated to ~400ºC during the destruction (which seems to be the case due to the results from the surrounding specimens and due to the temperature step at which the direction of the signal changes) ferromagnetic minerals with higher blocking temperatures maintained the original signal while minerals with lower blocking temperatures recorded the ambient field. In addition to SF09Q09t, two other specimens from SF09Q yielded two-component vectors (SF09Q15t and SF09Q39t). Also in these two cases, the components erased at higher temperatures met criteria (S9 Fig). The directions of the low-temperature components of these three specimens are roughly the same as all the other results of SF09Q. The directions of the high-temperature components of these three specimens are completely different from each other, proving that they do not originate in a previous heating event of the entire brick. This demonstrates once again the advantage of using oriented samples for temperature estimation and site formation analysis in general. To conclude, reconstructing the temperature from results which are not univectorial should be done with caution and, if possible, avoided.

FTIR spectra of the experimentally heated mudbrick material

As mentioned in the main text, up to 460ºC there were no significant changes in the FTIR spectra which could serve as an indication of fired clay (Fig 6B). Raising the temperature to 490ºC (Fig 6A) resulted in a spectrum that shows more significant changes: The Al-O-H absorption at ~914cm^{-1} and the bounded hydroxyls absorption at ~3691cm^{-1} are absent and the absorption at ~3620cm-^{1} is significantly reduced, as is the Si-O-Al absorption at ~518cm^{-1}. However, the Si-O-Si vibration at ~1033cm^{-1} has not shifted and appears the same as for the unheated clay. More significant changes are observed as the temperature

increases, corresponding to the breaking of the clay structure. The main observation is related to the Si-O-Si vibration which is generally shifted to higher wavenumbers as the temperature increases. At 520ºC the Si-O-Si absorption is shifted to ~1037cm^{-1}, at 550ºC to ~1041cm^{-1}, and at 640ºC to ~1049cm^{-1}. Also seen is a decrease in the 518cm^{-1} peak until it is totally lost at 700ºC. In this experiment, the shift of the Si-O-Si absorption in the 700ºC spectrum (~1041cm^{-1}) is smaller than that of the 640ºC spectrum and is thus inconsistent with the rise in temperature. Also noteworthy is that at this temperature the calcite peaks decrease, suggesting the beginning of dissociation of $CaCO_3$ to CaO. The shifts of the main clay absorption, which were considered reliable criteria for temperature estimations [4, 5] are not consistent at 640-700ºC [6].

References

1. Goodman-Elgar M. The devolution of mudbrick: ethnoarchaeology of abandoned earthen dwellings in the Bolivian Andes. Journal of Archaeological Science. 2008;35(12):3057-71.
2. Lorenzon M, Nitschke JL, Littman RJ, Silverstein JE. Mudbricks, Construction Methods, and Stratigraphic Analysis: A Case Study at Tell Timai (Ancient Thmuis) in the Egyptian Delta. American Journal of Archaeology. 2020;124(1):105-31.
3. Rosenberg D, Love S, Hubbard E, Klimscha F. 7,200 years old constructions and mudbrick technology: The evidence from Tel Tsaf, Jordan Valley, Israel. PLOS ONE. 2020;15(1):e0227288.
4. Shoval S, Erez Z, Kirsh Y, Deutsch Y, Kochavi M, Yadin E. Determination of the intensity of an early iron age conflagration at Tel-Hadar, Israel. Thermochimica Acta. 1989;148:485-92.
5. Berna F, Behar A, Shahack-Gross R, Berg J, Boaretto E, Gilboa A, et al. Sediments exposed to high temperatures: reconstructing pyrotechnological processes in Late Bronze and Iron Age Strata at Tel Dor (Israel). Journal of Archaeological Science. 2007;34(3):358-73.
6. Forget MCL, Regev L, Friesem DE, Shahack-Gross R. Physical and mineralogical properties of experimentally heated chaff-tempered mud bricks: Implications for reconstruction of environmental factors influencing the appearance of mud bricks in archaeological conflagration events. Journal of Archaeological Science: Reports. 2015;2:80-93.
